# Optimization of the cut configuration for skin grafts

**DOI:** 10.1007/s10237-025-02035-5

**Published:** 2026-01-04

**Authors:** Helmut Harbrecht, Viacheslav Karnaev

**Affiliations:** Departement Mathematik und Informatik, Universität Basel, 4051 Basel, Switzerland

**Keywords:** Skin grafts, Cut configuration, Optimization

## Abstract

The subject of this work is the problem of optimizing the configuration of cuts for skin grafting in order to improve the efficiency of the procedure. We consider the optimization problem in the framework of a linear elasticity model. We choose three mechanical measures that define optimality via related objective functionals: the compliance, the $L^p$-norm of the von Mises stress, and the area covered by the stretched skin. We provide a proof of the existence of the solution for each problem, but we cannot claim uniqueness. We compute the gradient of the objectives with respect to the cut configuration using concepts from shape calculus. To solve the problem numerically, we apply the gradient descent method, which performs well under uniaxial stretching. However, in more complex cases, such as multidirectional stretching, its effectiveness is limited due to the low sensitivity of the functionals under consideration.To avoid this difficulty, we use a combination of the genetic algorithm and the gradient descent method, which leads to a significant improvement in the results.

## Introduction

Skin grafting is a surgical procedure. Healthy skin is removed from one part of the body and transplanted. The healthy skin covers or replaces damaged or lost skin, for example, on the lower leg, to get a wound healed. The most commonly used skin graft is the *split-thickness skin graft* (SSG) which is a thin layer of shaved skin, see Maskan Bermudez et al. (2024); Taylor et al. (2021) for example. It is called *split-thickness* because only the epidermis and part of the dermis is shaved off, leaving part of the skin behind. As a result, the part left behind, called the *donor site*, can heal on its own without needing any additional skin covering.

To transplant as small a piece of skin as possible or, vice versa, in order to cover as large a piece of skin as possible, numerous parallel rows of short cuts are made in the healthy, harvested skin by using a suitable skin graft mesh device. The question we like to address in this article is: *Is there a better pattern to enlarge the healthy piece ﻿of skin than just making parallel cuts?* We tackle this problem by means of shape optimization (Delfour and Zolésio 2011; Henrot and Pierre 2018; Pironneau 1983; Sokołowski and Zolésio 1992), which means we are searching for an optimal layout of the cuts. To this end, we model the piece of skin by the equations of linear elasticity in two spatial dimensions and try to optimize the orientation of specific, predefined cuts such that a cost functional is optimized. We consider different cost functionals when stretching the skin: We (a) minimize the von Mises stress which ensures a small loading of the piece of skin, (b) maximize the area of the skin after the stretching procedure, and (c) minimize the compliance of the piece of skin. We analytically compute the Hadamard shape gradient for either cost functional which enables us to perform a gradient-based optimization algorithm.

Note that the linear-elastic model is the simplest constitutive model which is used to represent the mechanical behaviour of skin and its layers, see, e.g. Joodaki and Panzer (2018) for an overview of different skin models. Human skin is indeed not a simple object from the modelling point of view and its properties would be better described by a non-linear hyperelasticity model. However, we have chosen this simple model in order to be able to compute analytical expressions of the shape gradients of the cost functions under consideration. Moreover, the numerical simulation of the equations of linear elasticity can easily be carried out using the finite element method, compare (Braess 2001; Brenner and Scott 2008) for example. We use in our computations the finite element solver FreeFem++, see Hecht (2012).

Our particular setup is as follows. A quadratic piece of skin is subdivided into regular grid cells, each of which contains a cut that is anchored in the centre of the cell. The design variable is the rotation angle of the cut. However, it turns out that the shape functionals are not very sensitive with respect to the design variables. Moreover, we observe numerous local minima. We have therefore decided to group the cells into blocks that all match, so that the skin is represented by identical copies of one of these blocks. Moreover, we also combined the gradient-based optimization with a genetic algorithm in order to avoid to get stuck in a local minimum. Both ideas improve the optimization results considerably, as can be seen later in Sect. 4.

We finally like to mention related literature. An extensive study of different patterns in skin grafting is found in Gupta et al. (2022). Likewise, also in Capek et al. (2018); Gupta and Chanda (2023); Gupta et al. (2022) one can find results of forward simulations for predefined patterns. In contrast to these articles, however, we try to find the optimal pattern for skin grafting in case of the specific situation under consideration by an optimization algorithm. Indeed, we are only aware of Sutula et al. (2020), where also shape optimization has been exploited by means of a phase-field model in combination with a first discretize-then-optimize approach. Nonetheless, the results therein are completely different from our findings as we only vary the orientation of predefined cuts.

The rest of this article is organized as follows. In Sect. 2, we formulate the problem under consideration and cast it into shape optimization problems given by three different shape functionals. We then derive in Sect. 3 the related shape gradients and show the existence of solutions to the shape optimization problems under consideration. The numerical method is introduced in Sect. 4. Results of the numerical optimization process are presented which demonstrate the feasibility of our approach. Finally, concluding remarks are stated in Sect. 5.

## Problem formulation

We first introduce some general notation and define the governing equations describing a stretched elastic body with $N$ cuts modelled as holes with a prescribed shape, size, and position of the centres. Each hole is thus uniquely determined by a rotation angle. Hence, the configuration of the cuts is defined by $N$ angles. We proceed with the formulation of the shape optimization problems for the unknown angles in order to find the optimal configuration of the cuts. We finish this section by proving the existence of solutions to the optimization problems under consideration.

### Notation

Let $\Omega \subset \mathbb {R}^2$ be a bounded and connected domain with Lipschitz-smooth external boundary $\Gamma$ that is divided into two subsets $\Gamma _{{\mathcal {D}}}$ and $\Gamma _{{\mathcal {N}}}$ satisfying

$$\begin{aligned} |\Gamma _{{\mathcal {D}}}| > 0 \quad \text {such that} \quad \Gamma = \overline{\Gamma }_\mathcal {D} \cup \overline{\Gamma }_\mathcal {N} \quad \text {and}\quad \Gamma _{{\mathcal {D}}}\cap \Gamma _{{\mathcal {N}}}=\emptyset . \end{aligned}$$

We further assume that there are $N$ holes with smooth boundaries $\omega _{\alpha _i}, \ i=1,\dots ,N$, inside the domain. They are of the same shape and size, positioned on an equispaced grid, but with different angles of deviation from the abscissa axis. The set of all holes is denoted as

$$\begin{aligned} {\boldsymbol{\omega }}_{\boldsymbol{\alpha }}:= \bigcup _{i=1}^N \omega _{\alpha _i}, \quad \text {where}\quad \boldsymbol{\alpha }=(\alpha _1,\dots ,\alpha _N)\in [0,2\pi )^N. \end{aligned}$$

We shall consider an elastic body with *N* cuts, represented by the domain $\Omega _{\boldsymbol{\alpha }}:=\Omega {\setminus }{\boldsymbol{\omega }}_{\boldsymbol{\alpha }}$. The state of the body is determined by the vector field of displacements ${\boldsymbol{u}}:\Omega \rightarrow \mathbb {R}^2$. The mechanical properties of the body are completely characterized by the constant, symmetric, fourth-order stiffness tensor $\mathbb {C}$, which incorporates the material parameters—the Young modulus $E > 0$ and the Poisson ratio $-1< \nu < 1/2$.

Throughout this article, we use the notation

$$\begin{aligned} \varepsilon ({\boldsymbol{u}}):=\frac{1}{2}\left( \boldsymbol{\nabla }{\boldsymbol{u}}+\boldsymbol{\nabla }{\boldsymbol{u}}^\top \right) , \quad \text {where}\quad [\boldsymbol{\nabla }{\boldsymbol{u}}]_{i,j}:= \frac{\partial u_i}{\partial x_j}, \ i,j=1,2, \end{aligned}$$

for the deformation tensor and

$$\begin{aligned} \sigma ({\boldsymbol{u}}):= \mathbb {C}:\varepsilon ({\boldsymbol{u}}) = 2\mu \varepsilon ({\boldsymbol{u}}) + \lambda \text {div}({\boldsymbol{u}}){{{\,\mathrm{{{\textbf {I}}}}\,}}} \end{aligned}$$

for the stress tensor, where ${{{\,\mathrm{{{\textbf {I}}}}\,}}}$ is the $2\times 2$ identity matrix and the Lamé constants are

$$\begin{aligned} \mu =\frac{E}{2(1+\nu )} \quad \text {and} \quad \lambda =\frac{\nu E}{(1+\nu )(1-2\nu )}. \end{aligned}$$

### Governing equations

To simplify the model, we consider the cuts as thin, elliptical holes $\omega _{\alpha _i}$, $i=1,\dots ,N$. The main mechanical quantity is the displacement field ${\boldsymbol{u}}\in H^1(\Omega _{\boldsymbol{\alpha }})^2$. It is described by the equations of linear elasticity, supplemented by boundary conditions. The elastic body is subject to the body force $\boldsymbol{f} \in L^2(\Omega _{\boldsymbol{\alpha }})^2$ in the whole domain $\Omega _{\boldsymbol{\alpha }}$ and the external displacements $\boldsymbol{g} \in L^2(\Gamma _{{\mathcal {D}}})^2$ on the part $\Gamma _{{\mathcal {D}}}$ of its outer boundary, while the remaining part $\Gamma _{{\mathcal {N}}}$ and the boundaries $\partial \boldsymbol{\omega }_{\alpha }$ of the holes are unconstrained. Therefore, we arrive at the boundary value problem

2.1 $$\begin{aligned} \left\{ \; \begin{aligned} -\text {div}\big (\sigma ({\boldsymbol{u}})\big )&= \boldsymbol{f} \quad \text {in}\quad \Omega _{\boldsymbol{\alpha }}, \\ \sigma ({\boldsymbol{u}}) \boldsymbol{n}&= \boldsymbol{0}\quad \text {on}\quad \Gamma _{{\mathcal {N}}}\cup \partial \boldsymbol{\omega }_{\boldsymbol{\alpha }}, \\ {\boldsymbol{u}}&= \boldsymbol{g} \quad \text {on}\quad \Gamma _{{\mathcal {D}}}. \end{aligned} \right. \end{aligned}$$

**Fig. 1:**
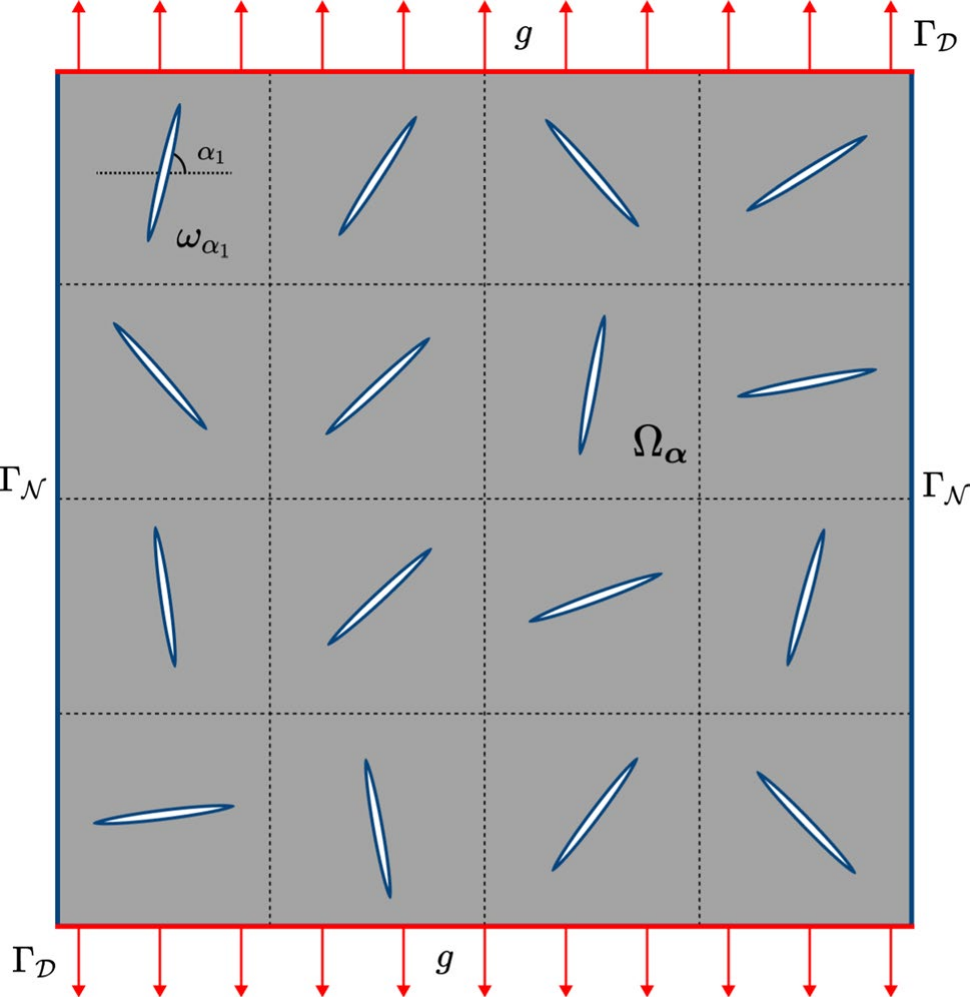
The model for the elastic body with cuts which are represented by thin elliptical holes

Here, $\boldsymbol{n}$ denotes the outward pointing unit normal vector on $\partial \Omega _{\boldsymbol{\alpha }}$. A visualization of the model can be found in Fig. 1.

In order to derive the variational formulation of (2.1), we define the space of admissible displacements

$$\begin{aligned} {\boldsymbol{H}}(\Omega _{\boldsymbol{\alpha }}):= \left\{ {\boldsymbol{u}}\in H^1(\Omega _{\boldsymbol{\alpha }})^2 \ | \ {\boldsymbol{u}}= \boldsymbol{g} \ \text {on} \ \Gamma _{{\mathcal {D}}}\right\} . \end{aligned}$$

Moreover, the respective test space is given by

$$\begin{aligned} {\boldsymbol{H}}_0(\Omega _{\boldsymbol{\alpha }}):= \left\{ {\boldsymbol{u}}\in H^1(\Omega _{\boldsymbol{\alpha }})^2 \ | \ {\boldsymbol{u}}= \boldsymbol{0}\ \text {on} \ \Gamma _{{\mathcal {D}}}\right\} . \end{aligned}$$

The scalar product and norm in ${\boldsymbol{H}}(\Omega _{\boldsymbol{\alpha }})$ defined as

2.2 $$\begin{aligned} \langle {\boldsymbol{u}},{\boldsymbol{v}}\rangle _{{\boldsymbol{H}}(\Omega _{\boldsymbol{\alpha }})} =\int _{\Omega _{\boldsymbol{\alpha }}}\sigma ({\boldsymbol{u}}): \varepsilon ({\boldsymbol{v}}){{\,\mathrm{\text {d}\,\,\!{\boldsymbol{x}}}\,}}\nonumber \\  \quad \text {and}\quad \Vert {\boldsymbol{u}}\Vert _{{\boldsymbol{H}}(\Omega _{\boldsymbol{\alpha }})} = \int _{\Omega _{\boldsymbol{\alpha }}}\sigma ({\boldsymbol{u}}): \varepsilon ({\boldsymbol{u}}){{\,\mathrm{\text {d}\,\,\!{\boldsymbol{x}}}\,}}. \end{aligned}$$

In the same way, it is defined in ${\boldsymbol{H}}_0(\Omega _{\boldsymbol{\alpha }})$. Thus, the variational formulation associated to the system (2.1) reads as follows: find ${\boldsymbol{u}}\in {\boldsymbol{H}}(\Omega _{\boldsymbol{\alpha }})$ such that

2.3 $$\begin{aligned} \int _{\Omega _{\boldsymbol{\alpha }}}\sigma ({\boldsymbol{u}}): \varepsilon ({\boldsymbol{v}}){{\,\mathrm{\text {d}\,\,\!{\boldsymbol{x}}}\,}}= \int _{\Omega _{\boldsymbol{\alpha }}} \boldsymbol{f}\cdot {\boldsymbol{v}}{{\,\mathrm{\text {d}\,\,\!{\boldsymbol{x}}}\,}}\quad \forall \,{\boldsymbol{v}}\in {\boldsymbol{H}}_0(\Omega _{\boldsymbol{\alpha }}). \end{aligned}$$

In view of the Lax–Milgram theorem together with Korn’s inequality, the variational formulation (2.3) and hence the system (2.1) admits a unique solution ${\boldsymbol{u}}\in {\boldsymbol{H}}(\Omega _{\boldsymbol{\alpha }})$.

### Optimization problem

We are looking for the optimal configuration $\Omega _{\boldsymbol{\alpha }}$ of the model described above. By optimal, we mean that the objective functional reaches a minimum or maximum (depending on the task at hand) in the set of admissible configurations. We consider two different cost functionals for the minimization problem, the compliance and the $L^p$-norm of the von Mises stress, and one for the maximization problem, the area of the deformed body. Here and in the following, we mean by ${\boldsymbol{u}}_{\boldsymbol{\alpha }}$ the solution of (2.1) for the body $\Omega _{\boldsymbol{\alpha }}$ that is determined by $\boldsymbol{\alpha }\in \mathbb {R}^N$.

- The compliance is given by
  $$\begin{aligned} \mathcal {C}(\Omega _{\boldsymbol{\alpha }}):= \int _{\Omega _{\boldsymbol{\alpha }}}\sigma ({\boldsymbol{u}}_{\boldsymbol{\alpha }}):\varepsilon ({\boldsymbol{u}}_{\boldsymbol{\alpha }}){{\,\mathrm{\text {d}\,\,\!{\boldsymbol{x}}}\,}}. \end{aligned}$$
- The von Mises stress in $\mathbb {R}^2$ is defined as
  $$\begin{aligned} \sigma _{\text {VM}}({\boldsymbol{u}}):= \sqrt{\sigma _d({\boldsymbol{u}}):\sigma _d({\boldsymbol{u}})}, \end{aligned}$$
  where $\sigma _d$ is the stress deviator tensor
  $$\begin{aligned} \sigma _d({\boldsymbol{u}}):= \sigma ({\boldsymbol{u}}) - \frac{\text {tr}(\sigma ({\boldsymbol{u}}))}{2}{{\,\mathrm{{{\textbf {I}}}}\,}}= 2\mu \varepsilon ({\boldsymbol{u}}) - \text {div}({\boldsymbol{u}}){{\,\mathrm{{{\textbf {I}}}}\,}}. \end{aligned}$$
  Of central interest from an application point of view is the $L^\infty$-norm of the von Mises stress. However, it is not differentiable. Therefore, since we want to use gradient-based methods, we consider the $L^p$-norm, which is known to converge to the $L^\infty$-norm at $p\rightarrow \infty$. So we define the objective functional as
  $$\begin{aligned} {{\,\mathrm{\mathcal {M}}\,}}(\Omega _{\boldsymbol{\alpha }}):  = \left( \int _{\Omega _{\boldsymbol{\alpha }}} |\sigma _{\text {VM}}({\boldsymbol{u}}_{\boldsymbol{\alpha }})|^p{{\,\mathrm{\text {d}\,\,\!{\boldsymbol{x}}}\,}}\right) ^{1/p} \\  = \left( \int _{\Omega _{\boldsymbol{\alpha }}} (\sigma _d({\boldsymbol{u}}_{\boldsymbol{\alpha }}):\sigma _d({\boldsymbol{u}}_{\boldsymbol{\alpha }}))^{p/2}{{\,\mathrm{\text {d}\,\,\!{\boldsymbol{x}}}\,}}\right) ^{1/p}. \end{aligned}$$
  Since we consider a convex domain with smooth cuts, we have ${\boldsymbol{u}}_{\boldsymbol{\alpha }}\in H^2(\Omega _{\boldsymbol{\alpha }})^2$. Thus, there holds $\boldsymbol{\nabla }{\boldsymbol{u}}_{\boldsymbol{\alpha }}\in H^1(\Omega _{\boldsymbol{\alpha }})^{2\times 2}\hookrightarrow L^q(\Omega _{\boldsymbol{\alpha }})^{2\times 2}$ for any finite $q$. As a consequence, ${{\,\mathrm{\mathcal {M}}\,}}(\Omega _{\boldsymbol{\alpha }})$ is well defined for any finite $p$.
- The deformed body is defined as $(\Omega _{\boldsymbol{\alpha }})_{{\boldsymbol{u}}_{\boldsymbol{\alpha }}}:=({{\,\mathrm{{{\textbf {Id}}}}\,}}+ {\boldsymbol{u}}_{\boldsymbol{\alpha }})(\Omega _{\boldsymbol{\alpha }})$, where ${{\,\mathrm{{{\textbf {Id}}}}\,}}: \mathbb {R}^2\rightarrow \mathbb {R}^2$ is the identity mapping. Thus, its area is given by
  $$\begin{aligned} {{\,\mathrm{\mathcal {A}}\,}}(\Omega _{\boldsymbol{\alpha }}):= \int _{(\Omega _{\boldsymbol{\alpha }})_{{\boldsymbol{u}}_{\boldsymbol{\alpha }}}}{{\,\mathrm{\text {d}\,\,\!{\boldsymbol{x}}}\,}}= \int _{\Omega _{\boldsymbol{\alpha }}}\det ({{\,\mathrm{{{\textbf {I}}}}\,}}+\boldsymbol{\nabla }{\boldsymbol{u}}_{\boldsymbol{\alpha }}){{\,\mathrm{\text {d}\,\,\!{\boldsymbol{x}}}\,}}. \end{aligned}$$
  We note that the change of the local measure of area in linearized elasticity is characterized by $\text {div}({\boldsymbol{u}}_{\boldsymbol{\alpha }})$, which corresponds to the first-order expansion of $\det ({{\,\mathrm{{{\textbf {I}}}}\,}}+\boldsymbol{\nabla }{\boldsymbol{u}}_{\boldsymbol{\alpha }})$. In order to keep the formulation consistent with possible extensions to finite (nonlinear) elasticity, we define the functional ${{\,\mathrm{\mathcal {A}}\,}}(\Omega _{\boldsymbol{\alpha }})$ by using the determinant. In the linear elasticity case considered in this article, both definitions coincide up to higher-order terms, while the chosen form allows for the seamless transition to large-deformation models.

We define the set of admissible configurations as

$$\begin{aligned} {\boldsymbol{O}}_{2\pi }:= \{\Omega _{\boldsymbol{\alpha }}\subset \mathbb {R}^2 \ | \ \boldsymbol{\alpha }\in (\mathbb {R}\ \text {mod} \ 2\pi )^N\}. \end{aligned}$$

Note that the set ${\boldsymbol{O}}_{2\pi }$ is homeomorphic to $(\mathbb {R}\ \text {mod} \ 2\pi )^N$ which is compact in the standard Euclidean metric $d_{\mathbb {R}^N}(\cdot ,\cdot )$. Thus, we conclude compactness of ${\boldsymbol{O}}_{2\pi }$ in the metric that is defined by

$$\begin{aligned} d(\Omega _{\boldsymbol{\alpha }}, \Omega _{\boldsymbol{\alpha }^*}):= d_{\mathbb {R}^N}\big (A^{-1}(\Omega _{\boldsymbol{\alpha }}),A^{-1}(\Omega _{\boldsymbol{\alpha }^*})\big ) = d_{\mathbb {R}^N}(\boldsymbol{\alpha },\boldsymbol{\alpha }^*), \end{aligned}$$

where $A: (\mathbb {R}\ \text {mod} \ 2\pi )^N \rightarrow {\boldsymbol{O}}_{2\pi }$ is a homeomorphism.

We are now in the position to formulate the three shape optimization problems for finding the optimal cuts in the body as follows: find $\Omega _{\boldsymbol{\alpha }_{\mathcal {C}}}, \Omega _{\boldsymbol{\alpha }_{{{\,\mathrm{\mathcal {M}}\,}}}}, \Omega _{\boldsymbol{\alpha }_{{{\,\mathrm{\mathcal {A}}\,}}}}\in {\boldsymbol{O}}_{2\pi }$ such that

2.4 $$\begin{aligned} \mathcal {C}(\Omega _{\boldsymbol{\alpha }_{\mathcal {C}}})&=\underset{\boldsymbol{\alpha }\in {\boldsymbol{O}}_{2\pi }}{\text {inf}} \quad \mathcal {C}(\Omega _{\boldsymbol{\alpha }}), \end{aligned}$$

2.5 $$\begin{aligned} {{\,\mathrm{\mathcal {M}}\,}}(\Omega _{\boldsymbol{\alpha }_{{{\,\mathrm{\mathcal {M}}\,}}}})&=\underset{\boldsymbol{\alpha }\in {\boldsymbol{O}}_{2\pi }}{\text {inf}} \quad {{\,\mathrm{\mathcal {M}}\,}}(\Omega _{\boldsymbol{\alpha }}), \end{aligned}$$

2.6 $$\begin{aligned} {{\,\mathrm{\mathcal {A}}\,}}( \Omega _{\boldsymbol{\alpha }_{{{\,\mathrm{\mathcal {A}}\,}}}})&=\underset{\boldsymbol{\alpha }\in {\boldsymbol{O}}_{2\pi }}{\text {sup}} \quad {{\,\mathrm{\mathcal {A}}\,}}(\Omega _{\boldsymbol{\alpha }}). \end{aligned}$$

Since these optimization problems are formulated over a compact set, we only need to show that the above functionals are continuous in order to prove the existence of a solution. However, there is a certain difficulty as the functionals depend not only directly on $\boldsymbol{\alpha }$, but also on the solution ${\boldsymbol{u}}_{\boldsymbol{\alpha }}$. The sequence $\{\Omega _{\boldsymbol{\alpha }_n}\}_{n\in \mathbb {N}}\subset {\boldsymbol{O}}_{2\pi }$ corresponds to the relative sequence of fields $\{{\boldsymbol{u}}_n\in {\boldsymbol{H}}(\Omega _{\boldsymbol{\alpha }_n})\}_{n\in \mathbb {N}}$, each element of which is defined in its own space. Therefore, for mathematical analysis, we will map them to a single, fixed space as follows.

Let us fix some arbitrary $\Omega _{\boldsymbol{\alpha }^*}\in {\boldsymbol{O}}_{2\pi }$ and a sequence $\{\Omega _{\boldsymbol{\alpha }_n}\}_{n\in \mathbb {N}}\subset {\boldsymbol{O}}_{2\pi }$. Since the cuts $\omega _{\alpha _{n,i}}$ do not intersect each other for fixed $n$, we can define the non-intersecting and closed sets $B_{r_i} := \{{\boldsymbol{x}}\in \mathbb {R}^2: \Vert {\boldsymbol{x}}\Vert _2\le r_i\}$ and $B_{R_i}\{{\boldsymbol{x}}\in \mathbb {R}^2: \Vert {\boldsymbol{x}}\Vert _2\le R_i\}$ such that $\omega _{\alpha _{n,i}}\subset B_{r_i} \subsetneq B_{R_i}\subset \Omega$ for any $n$. Let $\chi _i({\boldsymbol{x}})\in C_0^\infty (\Omega )$ be such that

2.7 $$\begin{aligned} \chi _i({\boldsymbol{x}}):= {\left\{ \begin{array}{ll} 1, \quad \text {if}\quad {\boldsymbol{x}}\in B_{r_i}, \\ 0, \quad \text {if}\quad {\boldsymbol{x}}\in \Omega \setminus B_{R_i}, \end{array}\right. } \end{aligned}$$

for $i=1,\dots ,N$. Let further $\{{\textbf {O}}_{i,n}\}_{n\in \mathbb {N}}$ be a sequence of orthogonal matrices defined as

$$\begin{aligned} & {\textbf {O}}_{n,i} = \begin{bmatrix} \cos (\alpha _i^*-\alpha _{n,i}) & -\sin (\alpha _i^*-\alpha _{n,i}) \\ \sin (\alpha _i^*-\alpha _{n,i}) & \phantom {-}\cos (\alpha _i^*-\alpha _{n,i}) \end{bmatrix} \quad \text {for any} \\ & i=1,\dots ,N\ \text {and}\ n\in \mathbb {N}. \end{aligned}$$

Thus, we define a sequence $\{\boldsymbol{\phi }_n\}_{n\in \mathbb {N}}$ of diffeomorphisms in accordance with

2.8 $$\begin{aligned} \boldsymbol{\phi }_n({\boldsymbol{x}}):= {\boldsymbol{x}}+ \sum _{i=1}^{N}\chi _i({\boldsymbol{x}})({\textbf {O}}_{i,n}{\boldsymbol{x}}-{\boldsymbol{x}}) \quad \text {for any}\ n\in \mathbb {N}. \end{aligned}$$

These mappings do not touch the outer boundary $\Gamma$. In particular, there holds $\boldsymbol{\phi }_n(\Omega _{\boldsymbol{\alpha }_n}) = \Omega _{\boldsymbol{\alpha }^*}$ and $\boldsymbol{\phi }_n^{-1}(\Omega _{\boldsymbol{\alpha }^*}) = \Omega _{\boldsymbol{\alpha }_n}$. Moreover, in the neighbourhood of an arbitrary cut $\omega _{\alpha _i}$, this mapping is expressed as $\boldsymbol{\phi }_n({\boldsymbol{x}})={\textbf {O}}_{i,n}{\boldsymbol{x}}$ and, in the neighbourhood of the external boundary, as $\boldsymbol{\phi }_n({\boldsymbol{x}})={\boldsymbol{x}}$. In addition, the Jacobi matrix of $\boldsymbol{\phi }_n$ is given by

2.9 $$\begin{aligned} \boldsymbol{\nabla }\boldsymbol{\phi }_n({\boldsymbol{x}})&:= {{\,\mathrm{{{\textbf {I}}}}\,}}+ {{\,\mathrm{{{\textbf {J}}}}\,}}_n({\boldsymbol{x}}) \nonumber \\&\quad \text {with}\quad {{\,\mathrm{{{\textbf {J}}}}\,}}_n({\boldsymbol{x}}) = \sum _{i=1}^{N}\boldsymbol{\nabla }\chi _i({\boldsymbol{x}})\otimes ({\textbf {O}}_{i,n}{\boldsymbol{x}}-{\boldsymbol{x}}) \nonumber \\&\quad \quad + \chi _i({\boldsymbol{x}})({\textbf {O}}_{i,n} - {{\,\mathrm{{{\textbf {I}}}}\,}}). \end{aligned}$$

For $n$ large enough, ${{\,\mathrm{{{\textbf {J}}}}\,}}_n$ becomes sufficiently close to zero, so $\det ({{\,\mathrm{{{\textbf {I}}}}\,}}+{{\,\mathrm{{{\textbf {J}}}}\,}}_n)>0$. Therefore, ${{\,\mathrm{{{\textbf {I}}}}\,}}+ {{\,\mathrm{{{\textbf {J}}}}\,}}_n$ remains invertible.

Introducing the mappings $\boldsymbol{\phi }_n$ resolves the aforementioned difficulty: by pulling back each solution to the fixed reference domain $\Omega _{\boldsymbol{\alpha }^*}$, i.e. ${\boldsymbol{u}}_{\boldsymbol{\alpha }_n}\circ \boldsymbol{\phi }_n^{-1}\in {\boldsymbol{H}}(\Omega _{\alpha _*})$, we place all objects in a single, fixed space and thus verify continuity of the functionals without comparing functions defined on different domains.

Before formulating the existence theorem, we need the following lemma and its proof.

#### Lemma 2.1

Assume $\Omega _{\boldsymbol{\alpha }^*}\in {\boldsymbol{O}}_{2\pi }$ and consider the sequence $\{\Omega _{\boldsymbol{\alpha }_n}\}_{n\in \mathbb {N}}\subset {\boldsymbol{O}}_{2\pi }$. Let ${\boldsymbol{u}}_n := {\boldsymbol{u}}_{\boldsymbol{\alpha }_n}$ for all $n\in \mathbb {N}$ and ${\boldsymbol{u}}^* := {\boldsymbol{u}}_{\boldsymbol{\alpha }^*}$ denote the corresponding solutions of (2.3) and define the mapped fields ${\widehat{{\boldsymbol{u}}}}_n := {\boldsymbol{u}}_n\circ \boldsymbol{\phi }_n\rightarrow {\boldsymbol{u}}^*$ via the mapping (2.8). If $\Omega _{\boldsymbol{\alpha }_n}\rightarrow \Omega _{\boldsymbol{\alpha }^*}$ as $n\rightarrow \infty$, then we have ${\widehat{{\boldsymbol{u}}}}_n \rightarrow {\boldsymbol{u}}^*$ strongly in ${\boldsymbol{H}}(\Omega _{\boldsymbol{\alpha }^*})$.

#### Proof

We can express the identity (2.3) in the form

$$\begin{aligned}\int _{\Omega _{\boldsymbol{\alpha }^*}}\mathbb {C}:\big (({{\,\mathrm{{{\textbf {I}}}}\,}}+ {{\,\mathrm{{{\textbf {J}}}}\,}}_n)^{-1}\boldsymbol{\nabla }{\widehat{{\boldsymbol{u}}}}_n\big ): \\\big (({{\,\mathrm{{{\textbf {I}}}}\,}}+ {{\,\mathrm{{{\textbf {J}}}}\,}}_n)^{-1}\boldsymbol{\nabla }{\widehat{{\boldsymbol{v}}}}\big )\det ({{\,\mathrm{{{\textbf {I}}}}\,}}+ {{\,\mathrm{{{\textbf {J}}}}\,}}_n){{\,\mathrm{\text {d}\,\,\!{\boldsymbol{x}}}\,}}\\= \int _{\Omega _{\boldsymbol{\alpha }^*}}(\boldsymbol{f}\cdot {\widehat{{\boldsymbol{v}}}})\det ({{\,\mathrm{{{\textbf {I}}}}\,}}+ {{\,\mathrm{{{\textbf {J}}}}\,}}_n){{\,\mathrm{\text {d}\,\,\!{\boldsymbol{x}}}\,}}\\\quad \forall {\boldsymbol{v}}\in {\boldsymbol{H}}_0(\Omega _{\boldsymbol{\alpha }^*}). \end{aligned}$$

Utilizing the Neumann series, i.e. the representation $({{\,\mathrm{{{\textbf {I}}}}\,}}+{{\,\mathrm{{{\textbf {J}}}}\,}}_n)^{-1} = {{\,\mathrm{{{\textbf {I}}}}\,}}- {{\,\mathrm{{{\textbf {J}}}}\,}}_n + {{\,\mathrm{{{\textbf {J}}}}\,}}_n^2 - \dots$, which is valid for $\Vert {{\,\mathrm{{{\textbf {J}}}}\,}}_n\Vert _2$ sufficiently small (as $n\rightarrow \infty$), and using the properties of determinants in $\mathbb {R}^2$ allows us to rewrite the expression as

2.10 $$\begin{aligned} \begin{aligned} \int _{\Omega _{\boldsymbol{\alpha }^*}} \sigma ({\widehat{{\boldsymbol{u}}}}_n): \varepsilon ({\widehat{{\boldsymbol{v}}}}){{\,\mathrm{\text {d}\,\,\!{\boldsymbol{x}}}\,}}&+ \mathcal {R}_n({\widehat{{\boldsymbol{u}}}}_n, {\widehat{{\boldsymbol{v}}}}) \\&= \int _{\Omega _{\boldsymbol{\alpha }^*}} \boldsymbol{f}\cdot {\widehat{{\boldsymbol{v}}}}{{\,\mathrm{\text {d}\,\,\!{\boldsymbol{x}}}\,}}\\&+ \int _{\Omega _{\boldsymbol{\alpha }^*}} (\boldsymbol{f} \cdot {\widehat{{\boldsymbol{v}}}}) \det ({{\,\mathrm{{{\textbf {J}}}}\,}}_n){{\,\mathrm{\text {d}\,\,\!{\boldsymbol{x}}}\,}}\\ \forall {\boldsymbol{v}}\in {\boldsymbol{H}}_0(\Omega _{\boldsymbol{\alpha }^*}).&\end{aligned} \end{aligned}$$

Herein, by the Cauchy–Schwarz inequality and the uniform boundedness of $\mathbb {C}$ and ${{\,\mathrm{{{\textbf {J}}}}\,}}_n$, the remainder term satisfies

$$\begin{aligned} |\mathcal {R}_n({\boldsymbol{u}}, {\boldsymbol{v}})| \le C \Vert {\textbf {O}}_{i,n} -{\textbf {I}}\Vert \Vert {\boldsymbol{u}}\Vert _{{\boldsymbol{H}}(\Omega _{\boldsymbol{\alpha }^*})}\Vert {\boldsymbol{v}}\Vert _{{\boldsymbol{H}}(\Omega _{\boldsymbol{\alpha }^*})} \quad \forall \ {\boldsymbol{u}},{\boldsymbol{v}}\in {\boldsymbol{H}}(\Omega _{\boldsymbol{\alpha }^*}) \end{aligned}$$

for some constant $C > 0$. In view of $\Omega _{\boldsymbol{\alpha }_n}\rightarrow \Omega _{\boldsymbol{\alpha }^*}$, by definition we get ${\textbf {O}}_{i,n} \rightarrow {\textbf {I}}$ and $\det ({{\,\mathrm{{{\textbf {J}}}}\,}}_n)\rightarrow 0$ for any $i=1,\dots ,N$ as $n\rightarrow \infty$. Thus, we conclude that $\mathcal {R}_n({\widehat{{\boldsymbol{u}}}}_n, {\widehat{{\boldsymbol{v}}}})\rightarrow 0$ for any ${\widehat{{\boldsymbol{v}}}}\in {\boldsymbol{H}}_0(\Omega _{\boldsymbol{\alpha }^*})$ as $n\rightarrow \infty$. Hence, we get

2.11 $$\begin{aligned} \int _{\Omega _{\boldsymbol{\alpha }^*}}\sigma ({\widehat{{\boldsymbol{u}}}}_n): \varepsilon ({\widehat{{\boldsymbol{v}}}}){{\,\mathrm{\text {d}\,\,\!{\boldsymbol{x}}}\,}}\rightarrow \int _{\Omega _{\boldsymbol{\alpha }^*}}\sigma ({\widetilde{{\boldsymbol{u}}}}): \varepsilon ({\widehat{{\boldsymbol{v}}}}){{\,\mathrm{\text {d}\,\,\!{\boldsymbol{x}}}\,}}\quad \forall \ {\widehat{{\boldsymbol{v}}}}\in {\boldsymbol{H}}(\Omega _{\boldsymbol{\alpha }^*}) \end{aligned}$$

as $n\rightarrow \infty$. Combining (2.10) and (2.11), we conclude that

$$\begin{aligned} \int _{\Omega _{\boldsymbol{\alpha }^*}}\sigma ({\widetilde{{\boldsymbol{u}}}}): \varepsilon ({\widehat{{\boldsymbol{v}}}}){{\,\mathrm{\text {d}\,\,\!{\boldsymbol{x}}}\,}}= \int _{\Omega _{\boldsymbol{\alpha }^*}}\boldsymbol{f}\cdot {\widehat{{\boldsymbol{v}}}}{{\,\mathrm{\text {d}\,\,\!{\boldsymbol{x}}}\,}}\quad \forall \,{\widehat{{\boldsymbol{v}}}}\in {\boldsymbol{H}}_0(\Omega _{\boldsymbol{\alpha }^*}). \end{aligned}$$

Since the solution of (2.3) is unique for each $\Omega _{\boldsymbol{\alpha }}$, we arrive at ${\widetilde{{\boldsymbol{u}}}}={\boldsymbol{u}}^*$. By the definition of the scalar product $\langle \cdot , \cdot \rangle _{{\boldsymbol{H}}(\Omega _{\boldsymbol{\alpha }^*})}$ in (2.2) and using (2.11), we deduce the weak convergence ${\widehat{{\boldsymbol{u}}}}_n \rightharpoonup {\boldsymbol{u}}^*$ in ${\boldsymbol{H}}(\Omega _{\boldsymbol{\alpha }^*})$, i.e.

$$\begin{aligned} \langle {\widehat{{\boldsymbol{u}}}}_n,{\widehat{{\boldsymbol{v}}}}\rangle _{{\boldsymbol{H}}(\Omega _{\boldsymbol{\alpha }^*})}&= \int _{\Omega _{\boldsymbol{\alpha }^*}}\sigma ({\widehat{{\boldsymbol{u}}}}_n): \varepsilon ({\widehat{{\boldsymbol{v}}}}){{\,\mathrm{\text {d}\,\,\!{\boldsymbol{x}}}\,}}\\&\rightarrow \int _{\Omega _{\boldsymbol{\alpha }^*}}\sigma ({\widetilde{{\boldsymbol{u}}}}): \varepsilon ({\widehat{{\boldsymbol{v}}}}){{\,\mathrm{\text {d}\,\,\!{\boldsymbol{x}}}\,}}\\&=\langle {\boldsymbol{u}}^*,{\widehat{{\boldsymbol{v}}}}\rangle _{{\boldsymbol{H}}(\Omega _{\boldsymbol{\alpha }^*})} \\&\quad \forall {\boldsymbol{v}}\in {\boldsymbol{H}}_0(\Omega _{\boldsymbol{\alpha }^*}). \end{aligned}$$

Inserting the test function ${\widehat{{\boldsymbol{v}}}} = {\widehat{{\boldsymbol{u}}}}_n$ in (2.10) and exploiting the weak convergence, we obtain

2.12 $$\begin{aligned} \begin{aligned} \int _{\Omega _{\boldsymbol{\alpha }^*}}\sigma ({\widehat{{\boldsymbol{u}}}}_n): \varepsilon ({\widehat{{\boldsymbol{u}}}}_n){{\,\mathrm{\text {d}\,\,\!{\boldsymbol{x}}}\,}}&+ \mathcal {R}_n({\widehat{{\boldsymbol{u}}}}_n, {\widehat{{\boldsymbol{u}}}}_n) \\&= \int _{\Omega _{\boldsymbol{\alpha }^*}}\boldsymbol{f} \cdot {\widehat{{\boldsymbol{u}}}}_n{{\,\mathrm{\text {d}\,\,\!{\boldsymbol{x}}}\,}}\\&\quad + \int _{\Omega _{\boldsymbol{\alpha }^*}}(\boldsymbol{f} \cdot {\widehat{{\boldsymbol{u}}}}_n)\det ({{\,\mathrm{{{\textbf {J}}}}\,}}_n){{\,\mathrm{\text {d}\,\,\!{\boldsymbol{x}}}\,}}\\&\rightarrow \int _{\Omega _{\boldsymbol{\alpha }^*}}\boldsymbol{f} \cdot {\boldsymbol{u}}^*{{\,\mathrm{\text {d}\,\,\!{\boldsymbol{x}}}\,}}. \end{aligned} \end{aligned}$$

On the other hand, we have

2.13 $$\begin{aligned} \int _{\Omega _{\boldsymbol{\alpha }^*}}\boldsymbol{f} \cdot {\boldsymbol{u}}^*{{\,\mathrm{\text {d}\,\,\!{\boldsymbol{x}}}\,}}= \int _{\Omega _{\boldsymbol{\alpha }^*}}\sigma ({\boldsymbol{u}}^*): \varepsilon ({\boldsymbol{u}}^*){{\,\mathrm{\text {d}\,\,\!{\boldsymbol{x}}}\,}}. \end{aligned}$$

Using the definition of the norm $\Vert \cdot \Vert _{{\boldsymbol{H}}(\Omega _{\boldsymbol{\alpha }^*})}$ from (2.2), and combining (2.12) with (2.13), we deduce the convergence

$$\begin{aligned} \Vert {\widehat{{\boldsymbol{u}}}}_n\Vert _{{\boldsymbol{H}}(\Omega _{\boldsymbol{\alpha }^*})}= & \int _{\Omega _{\boldsymbol{\alpha }^*}}\sigma ({\widehat{{\boldsymbol{u}}}}_n): \\ & \varepsilon 
({\widehat{{\boldsymbol{u}}}}_n){{\,\mathrm{\text 
{d}\,\,\!{\boldsymbol{x}}}\,}}\rightarrow \int _{\Omega _{\boldsymbol{\alpha }^*}}\sigma ({\boldsymbol{u}}^*): \varepsilon ({\boldsymbol{u}}^*){{\,\mathrm{\text {d}\,\,\!{\boldsymbol{x}}}\,}}= \Vert {\boldsymbol{u}}^*\Vert _{{\boldsymbol{H}}(\Omega _{\boldsymbol{\alpha }^*})}. \end{aligned}$$

Finally, combining the weak convergence ${\widehat{{\boldsymbol{u}}}}_n \rightharpoonup {\boldsymbol{u}}^*$ and the convergence $\Vert {\widehat{{\boldsymbol{u}}}}_n\Vert _{{\boldsymbol{H}}(\Omega _{\boldsymbol{\alpha }^*})} \rightarrow \Vert {\boldsymbol{u}}^*\Vert _{{\boldsymbol{H}}(\Omega _{\boldsymbol{\alpha }^*})}$, we conclude the strong convergence ${\widehat{{\boldsymbol{u}}}}_n \rightarrow {\boldsymbol{u}}^*$ in ${\boldsymbol{H}}(\Omega _{\boldsymbol{\alpha }^*})$. $\square$

#### Theorem 2.2

Each of the problems (2.4)–(2.6) allows its own solution.

#### Proof

For the sake of brevity, we present the proof only for the compliance $\mathcal {C}(\Omega _{\boldsymbol{\alpha }})$. In complete analogy, we can get the result for the shape functionals $\mathcal {M}(\Omega _{\boldsymbol{\alpha }})$ and $\mathcal {M}(\Omega _{\boldsymbol{\alpha }})$, respectively.

Let $\{\Omega _{\boldsymbol{\alpha }_n}\}_{n\in \mathbb {N}}\subset {\boldsymbol{O}}_{2\pi }$ and $\Omega _{\boldsymbol{\alpha }^*}\in {\boldsymbol{O}}_{2\pi }$ such that $\Omega _{\boldsymbol{\alpha }_n}\rightarrow \Omega _{\boldsymbol{\alpha }^*}$ as $n\rightarrow \infty$. Using the mappings defined in (2.8), we rewrite the functional $\mathcal {C}(\Omega _{\boldsymbol{\alpha }_n})$ as an integral over the domain $\Omega _{\boldsymbol{\alpha }^*}$ as

$$\begin{aligned} \mathcal {C}(\Omega _{\boldsymbol{\alpha }_n})&= \int _{\Omega _{\boldsymbol{\alpha }_n}}\sigma ({\boldsymbol{u}}_n): \varepsilon ({\boldsymbol{u}}_n){{\,\mathrm{\text {d}\,\,\!{\boldsymbol{x}}}\,}}\\&= \int _{\Omega _{\boldsymbol{\alpha }^*}}\mathbb {C}:\big (({{\,\mathrm{{{\textbf {I}}}}\,}}+ {{\,\mathrm{{{\textbf {J}}}}\,}}_n)^{-1}\boldsymbol{\nabla }{\widehat{{\boldsymbol{u}}}}_n\big ): \big (({{\,\mathrm{{{\textbf {I}}}}\,}}+ {{\,\mathrm{{{\textbf {J}}}}\,}}_n)^{-1}\boldsymbol{\nabla }{\widehat{{\boldsymbol{u}}}}_n\big )\\&\quad \cdot \det ({{\,\mathrm{{{\textbf {I}}}}\,}}+ {{\,\mathrm{{{\textbf {J}}}}\,}}_n){{\,\mathrm{\text {d}\,\,\!{\boldsymbol{x}}}\,}}. \end{aligned}$$

From Lemma 2.1, we thus conclude that

$$\begin{aligned} \mathcal {C}(\Omega _{\boldsymbol{\alpha }_n})&= \int _{\Omega _{\boldsymbol{\alpha }^*}}\mathbb {C}:\big (({{\,\mathrm{{{\textbf {I}}}}\,}}+ {{\,\mathrm{{{\textbf {J}}}}\,}}_n)^{-1}\boldsymbol{\nabla }{\widehat{{\boldsymbol{u}}}}_n\big ):\big (({{\,\mathrm{{{\textbf {I}}}}\,}}+ {{\,\mathrm{{{\textbf {J}}}}\,}}_n)^{-1}\boldsymbol{\nabla }{\widehat{{\boldsymbol{u}}}}_n\big )\\&\quad \det ({{\,\mathrm{{{\textbf {I}}}}\,}}+ {{\,\mathrm{{{\textbf {J}}}}\,}}_n){{\,\mathrm{\text {d}\,\,\!{\boldsymbol{x}}}\,}}\\&\rightarrow \int _{\Omega _{\boldsymbol{\alpha }^*}}\sigma ({\boldsymbol{u}}^*): \varepsilon ({\boldsymbol{u}}^*){{\,\mathrm{\text {d}\,\,\!{\boldsymbol{x}}}\,}}= \mathcal {C}(\Omega _{\boldsymbol{\alpha }^*}). \end{aligned}$$

By the extreme value theorem, every continuous functional on a compact set attains its extrema. Hence, since $\mathcal {C}$ is continuous and ${\boldsymbol{O}}_{2\pi }$ is compact, the desired claim follows. $\square$

#### Remark 2.3

We cannot guarantee the uniqueness of the minimizer as the problem under consideration may admit many local minima, in particular due to symmetries. For example, if the external displacements and admissible cuts are invariant under reflections or rotations, different but symmetric cut patterns give the same objective value. This is particularly observed in our numerical results in Subsect. 4.4.

## Sensitivity analysis

The most common way to solve optimization problems numerically is to use gradient-based algorithms, for example gradient descent or the quasi-Newton method. To define the gradient, we use the concept of shape calculus. We begin with an introduction of the basic notations and definitions, and finish with the expression of the shape gradient for each of the considered functionals. For a general overview of shape calculus, we refer the reader, for example, to Allaire et al. (2021); Henrot and Pierre (2018); Plotnikov and Sokolowski (2023); Sokołowski and Zolésio (1992).

### Fundamentals of shape calculus

Let us consider an arbitrary shape functional ${{\,\mathrm{\mathcal {F}}\,}}: {\boldsymbol{O}}_{2\pi }\rightarrow \mathbb {R}$ and perturbation fields $\boldsymbol{\theta }_i\in W^{1,\infty }(\Omega _{\boldsymbol{\alpha }})^2$ for $i=1,\dots ,N$ such that $\boldsymbol{\theta }_i({\boldsymbol{x}}) = 0$ if ${\boldsymbol{x}}\in \Gamma \cup \partial (\boldsymbol{\omega }_{\boldsymbol{\alpha }}{\setminus }\omega _i)$, i.e. it only perturbs the boundary of the respective cut. Then, for a sufficiently small parameter $\epsilon >0$, we define the perturbed body as

$$\begin{aligned} \Omega _{\boldsymbol{\alpha }_{\epsilon _i}}:= ({{\,\mathrm{{{\textbf {Id}}}}\,}}+ \epsilon \boldsymbol{\theta }_i)(\Omega _{\boldsymbol{\alpha }}). \end{aligned}$$

Since we consider cuts of fixed shape and size, the only thing we can perturb is their angles. So we define $\boldsymbol{\theta }_i$ as

3.1 $$\begin{aligned} \boldsymbol{\theta }_i({\boldsymbol{x}}):= \chi _i({\boldsymbol{x}})\begin{bmatrix} -x_2 \\ \phantom {+}x_1 \end{bmatrix}, \end{aligned}$$

where $\chi _i$ defined in (2.7). The whole perturbation in the neighbourhood of the cut is expressed as

$$\begin{aligned} ({{\,\mathrm{{{\textbf {Id}}}}\,}}+ \epsilon \boldsymbol{\theta }_i)({\boldsymbol{x}}) = \begin{bmatrix} 1 & -\epsilon \\ \epsilon & \phantom {+}1 \end{bmatrix} \begin{bmatrix} x_1 \\ x_2 \end{bmatrix} \quad \text {if}\quad {\boldsymbol{x}}\in B_{r_i}, \end{aligned}$$

which defines a linearized $\epsilon$ angle rotation, see Karnaev (2022) for the linearization. In the rest of the domain, the perturbation is the identity mapping

$$\begin{aligned} ({{\,\mathrm{{{\textbf {Id}}}}\,}}+ \epsilon \boldsymbol{\theta }_i)({\boldsymbol{x}}) = {{\,\mathrm{{{\textbf {Id}}}}\,}}\quad \text {if}\quad {\boldsymbol{x}}\in \Omega \setminus B_{R_i}. \end{aligned}$$

**Fig. 2:**
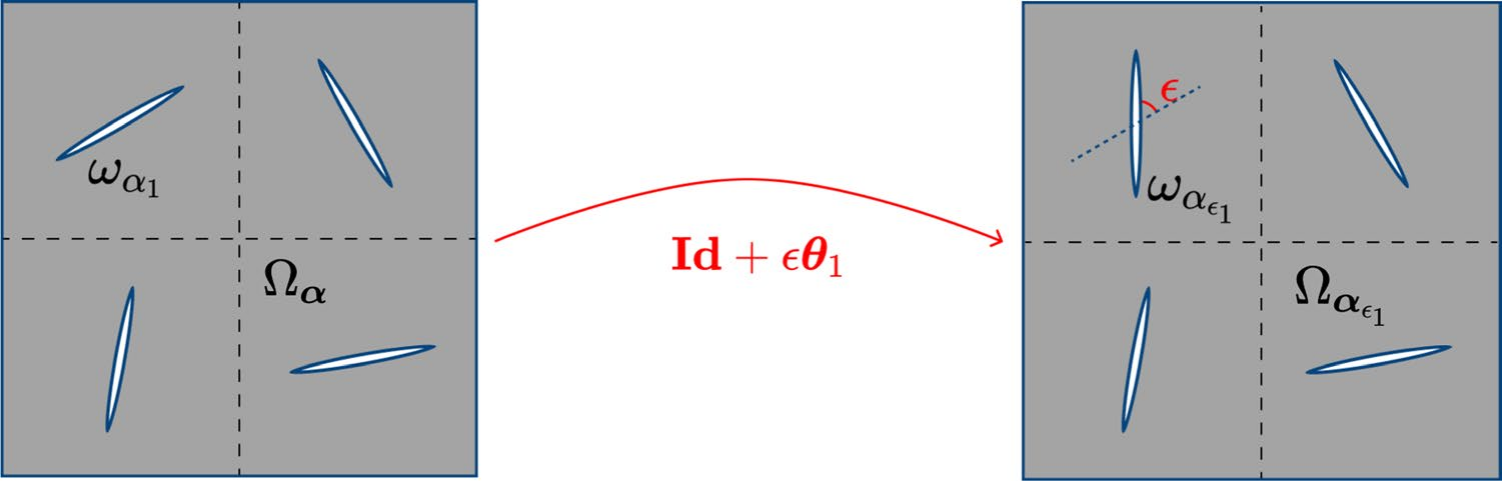
Variation $\Omega _{\boldsymbol{\alpha }_{\epsilon _1}}$of a shape $\Omega _{\boldsymbol{\alpha }}$ according to a deformation field $\boldsymbol{\theta }_1$

We refer to Fig. 2 for an illustration.

We can define the shape derivative of the functional along the direction given by the perturbation as follows.

#### Definition 3.1

The **shape derivative** of the shape functional ${{\,\mathrm{\mathcal {F}}\,}}$ is defined as the Gâteaux derivative at $\Omega _{\boldsymbol{\alpha }}$ in direction of the vector field $\boldsymbol{\theta }_i$, $i=1,\dots ,N$, i.e.

$$\begin{aligned} \text {d}\,\,\!{{\,\mathrm{\mathcal {F}}\,}}(\Omega _{\boldsymbol{\alpha }})\langle \boldsymbol{\theta }_i\rangle := \frac{\text {d}\,\,\!}{\text {d}\,\,\!\epsilon }{{\,\mathrm{\mathcal {F}}\,}}(({{\,\mathrm{{{\textbf {Id}}}}\,}}+ \epsilon \boldsymbol{\theta }_i)(\Omega _{\boldsymbol{\alpha }}))\Big |_{\epsilon =0}. \end{aligned}$$

Thus, we define the **shape gradient** as

$$\begin{aligned} & {{\,\mathrm{{{\textbf {D}}}\!}\,}}{{\,\mathrm{\mathcal {F}}\,}}(\Omega _{\boldsymbol{\alpha }})\langle \boldsymbol{\Theta }\rangle := \big [\text {d}\,\,\!{{\,\mathrm{\mathcal {F}}\,}}(\Omega _{\boldsymbol{\alpha }})\langle \boldsymbol{\theta }_1\rangle , \dots , \text {d}\,\,\!{{\,\mathrm{\mathcal {F}}\,}}(\Omega _{\boldsymbol{\alpha }})\langle \boldsymbol{\theta }_N\rangle \big ]^\top \quad \text {where} \\ & \quad \boldsymbol{\Theta }= [\boldsymbol{\theta }_1, \dots , \boldsymbol{\theta }_N]. \end{aligned}$$

In addition, we introduce the derivative of the displacement field ${\boldsymbol{u}}_{\boldsymbol{\alpha }}$, which quantifies the sensitivity of the solution to (2.1) with respect to variations in the domain. We follow the Lagrangian approach and introduce the following definition.

#### Definition 3.2

The **Lagrangian derivative** with respect to the perturbation field $\boldsymbol{\theta }_i$, $i=1,\dots ,N$, of the displacement field ${\boldsymbol{u}}_{\boldsymbol{\alpha }}$ at the domain $\Omega _{\boldsymbol{\alpha }}$ is defined as the Fréchet derivative of the transported solution at $\epsilon =0$, i.e.

$$\begin{aligned} \dot{{\boldsymbol{u}}}_{\boldsymbol{\alpha }}\langle \boldsymbol{\theta }_i\rangle := \left. \frac{\textrm{d}}{\textrm{d}\epsilon } \big ({\boldsymbol{u}}_{\boldsymbol{\alpha }_{\epsilon _i}}\circ ({{\,\mathrm{{{\textbf {Id}}}}\,}}+\epsilon \boldsymbol{\theta }_i)\big ) \right| _{\epsilon =0}, \end{aligned}$$

where ${\boldsymbol{u}}_{\boldsymbol{\alpha }_{\epsilon _i}}$ denotes the solution of (2.1) in the perturbed domain $\Omega _{\boldsymbol{\alpha }_{\epsilon _i}}$.

In the context of unconstrained shape optimization, the shape derivative is used to identify a direction $\boldsymbol{\theta }$ of deformation such that $\text {d}\,\,\!{{\,\mathrm{\mathcal {F}}\,}}(\Omega _{\boldsymbol{\alpha }})(\boldsymbol{\theta })< 0$. This direction of deformation serves as a descent direction in an appropriate optimization algorithm, which allows the minimization of the objective functional ${{\,\mathrm{\mathcal {F}}\,}}(\Omega _{\boldsymbol{\alpha }})$. Before proceeding to the shape derivatives of the functionals under consideration, we need to note that the shape derivative can be expressed via the integral over the boundary of the domain.

#### Remark 3.3

In the case of a sufficiently regular domain $\Omega _{\boldsymbol{\alpha }}$, due to Hadamard’s structure theorem (see, e.g. Henrot and Pierre (2018); Sokołowski and Zolésio (1992)), we can conclude that the value of the derivative $\text {d}\,\,\!{{\,\mathrm{\mathcal {F}}\,}}(D)\langle \cdot \rangle$ depends only on the normal component of the vector field $\boldsymbol{\theta }$ at the boundary $\partial \Omega _{\boldsymbol{\alpha }}$, i.e.

$$\begin{aligned} \text {d}\,\,\!{{\,\mathrm{\mathcal {F}}\,}}(\Omega _{\boldsymbol{\alpha }})\langle \boldsymbol{\theta }\rangle = \int _{\partial \Omega _{\boldsymbol{\alpha }}}v_{\Omega _{\boldsymbol{\alpha }}}(\boldsymbol{\theta }\cdot \boldsymbol{n}){{\,\mathrm{\text {d}\,\,\!\boldsymbol{s}}\,}}. \end{aligned}$$

Here, $v_{\Omega _{\boldsymbol{\alpha }}}:\partial \Omega _{\boldsymbol{\alpha }}\rightarrow \mathbb {R}$ is a scalar field whose expression depends on the solutions of the underlying boundary value problem and the functional. The expression is commonly called the **Hadamard’s form** of the shape derivative.

### Shape derivatives

We provide next statements about the boundary value problems underlying for the Lagrangian derivative of the displacement field ${\boldsymbol{u}}_{\boldsymbol{\alpha }}$ and about the expressions for the shape derivatives of functionals $\mathcal {C}(\Omega _{\boldsymbol{\alpha }})$ and ${{\,\mathrm{\mathcal {M}}\,}}(\Omega _{\boldsymbol{\alpha }})$. We do not give proofs of these statements since they are derived by standard techniques found for example in Allaire et al. (2021).

The next lemma characterizes the Lagrangian derivative of the displacement field ${\boldsymbol{u}}_{\boldsymbol{\alpha }}$.

#### Lemma 3.4

The Lagrangian derivative ${\dot{{\boldsymbol{u}}}}\in H^1(\Omega _{\boldsymbol{\alpha }})^2$ of the solution to (2.1) satisfies the boundary value problem

3.2 $$\begin{aligned} \left\{ \; \begin{aligned}&\text {div}(\sigma (\dot{{\boldsymbol{u}}}_{\boldsymbol{\alpha }}\langle \boldsymbol{\theta }_i\rangle ) = \text {div}\Big ((\mathbb {C}: \boldsymbol{\nabla }{\boldsymbol{u}}_{\boldsymbol{\alpha }})\boldsymbol{\nabla }\boldsymbol{\theta }^\top + \mathbb {C}:(\boldsymbol{\nabla }\boldsymbol{\theta }_i\boldsymbol{\nabla }{\boldsymbol{u}}_{\boldsymbol{\alpha }})&\\&\hspace{66mm} - \text {div}(\boldsymbol{\theta }_i)\,\sigma ({\boldsymbol{u}}_{\boldsymbol{\alpha }}) - \boldsymbol{f}\otimes \boldsymbol{\theta }_i\Big )\quad \text {in}\quad \Omega _{\boldsymbol{\alpha }}, \\&\sigma (\dot{{\boldsymbol{u}}}_{\boldsymbol{\alpha }})\boldsymbol{n} = \Big ((\mathbb {C}: \boldsymbol{\nabla }{\boldsymbol{u}}_{\boldsymbol{\alpha }})\boldsymbol{\nabla }\boldsymbol{\theta }^\top + \mathbb {C}:(\boldsymbol{\nabla }\boldsymbol{\theta }\boldsymbol{\nabla }{\boldsymbol{u}}_{\boldsymbol{\alpha }}) - \text {div}(\boldsymbol{\theta }_i)\sigma ({\boldsymbol{u}}_{\boldsymbol{\alpha }})\Big )\boldsymbol{n} \quad \text {on}\quad \partial \omega _i, \\&\sigma (\dot{{\boldsymbol{u}}}_{\boldsymbol{\alpha }})\boldsymbol{n} = \boldsymbol{0}\quad \text {on} \quad \Gamma _{{\mathcal {N}}}\cup \partial (\boldsymbol{\omega }_{\boldsymbol{\alpha }}\setminus \omega _i), \\&\dot{{\boldsymbol{u}}}_{\boldsymbol{\alpha }}= \boldsymbol{0}\hspace{13mm}\text {on}\quad \Gamma _{{\mathcal {D}}}, \end{aligned} \right. \end{aligned}$$

for any $i=1,\dots ,N$. It corresponds to the variational formulation

3.3 $$\begin{aligned} \begin{aligned}&\int _{\Omega _{\boldsymbol{\alpha }}}\sigma (\dot{{\boldsymbol{u}}}_{\boldsymbol{\alpha }}\langle \boldsymbol{\theta }_i\rangle ):\varepsilon ({\boldsymbol{v}}) = \int _{\Omega _{\boldsymbol{\alpha }}}\big (\mathbb {C}:(\boldsymbol{\nabla }\boldsymbol{\theta }_i\boldsymbol{\nabla }{\boldsymbol{u}}_{\boldsymbol{\alpha }}): \boldsymbol{\nabla }{\boldsymbol{v}}\\&\quad + \mathbb {C}:\boldsymbol{\nabla }{\boldsymbol{u}}_{\boldsymbol{\alpha }}:(\boldsymbol{\nabla }\boldsymbol{\theta }_i\boldsymbol{\nabla }{\boldsymbol{v}})\big ){{\,\mathrm{\text {d}\,\,\!{\boldsymbol{x}}}\,}}\\&-\int _{\Omega _{\boldsymbol{\alpha }}}\big (\text {div}(\boldsymbol{\theta }_i)\sigma ({\boldsymbol{u}}_{\boldsymbol{\alpha }}): \varepsilon ({\boldsymbol{v}}) - \text {div}(\boldsymbol{f}\otimes \boldsymbol{\theta }_i)\cdot {\boldsymbol{v}}\big ){{\,\mathrm{\text {d}\,\,\!{\boldsymbol{x}}}\,}}\\&\forall \,{\boldsymbol{v}}\in {\boldsymbol{H}}_0(\Omega _{\boldsymbol{\alpha }}). \end{aligned} \end{aligned}$$

The shape derivative of the compliance $\mathcal {C}(\Omega _{\boldsymbol{\alpha }})$ is presented in the next proposition.

#### Proposition 3.5

The shape derivative of $\mathcal {C}(\Omega _{\boldsymbol{\alpha }})$ is given by

3.4 $$\begin{aligned} \text {d}\,\,\!\mathcal {C}(\Omega _{\boldsymbol{\alpha }})\langle \boldsymbol{\theta }_i\rangle= & \int _{\partial \omega _i} \big (\sigma ({\boldsymbol{u}}_{\boldsymbol{\alpha }}):\varepsilon ({\boldsymbol{u}}_{\boldsymbol{\alpha }})\big ) \nonumber \\ & \big (\boldsymbol{\theta }_i\cdot \boldsymbol{n}\big ){{\,\mathrm{\text {d}\,\,\!\boldsymbol{s}}\,}}, \end{aligned}$$

where $$i=1,\dots ,N$$.

The shape derivative of the $L^p$-norm of the von Mises stress ${{\,\mathrm{\mathcal {M}}\,}}(\Omega _{\boldsymbol{\alpha }})$ is provided in the following proposition, see also Caubet et al. (2023).

#### Proposition 3.6

The shape derivative ${{\,\mathrm{\mathcal {M}}\,}}(\Omega _{\boldsymbol{\alpha }})$ is given by

3.5 $$\begin{aligned} \begin{aligned}&\text {d}\,\,\!{{\,\mathrm{\mathcal {M}}\,}}(\Omega _{\boldsymbol{\alpha }})\langle \boldsymbol{\theta }_i\rangle = \frac{1}{p}\bigg (\int _{\omega _i} \big ((\sigma _d({\boldsymbol{u}}_{\boldsymbol{\alpha }}) \\&:\sigma _d({\boldsymbol{u}}_{\boldsymbol{\alpha }}))^{p/2} - \sigma (\boldsymbol{p}_{\boldsymbol{\alpha }}):\varepsilon ({\boldsymbol{u}}_{\boldsymbol{\alpha }}) \\&+ (\boldsymbol{f}\cdot \boldsymbol{p}_{\boldsymbol{\alpha }})\big )\big (\boldsymbol{\theta }\cdot \boldsymbol{n}\big ){{\,\mathrm{\text {d}\,\,\!\boldsymbol{s}}\,}}\bigg )\left( \int _{\Omega _{\boldsymbol{\alpha }}} (\sigma _d({\boldsymbol{u}}_{\boldsymbol{\alpha }})\right. \\&\left. :\sigma _d({\boldsymbol{u}}_{\boldsymbol{\alpha }}))^{p/2}{{\,\mathrm{\text {d}\,\,\!{\boldsymbol{x}}}\,}}\right) ^{1/p - 1}, \end{aligned} \end{aligned}$$

where $i=1,\dots ,N$ and $\boldsymbol{p}_{\boldsymbol{\alpha }}\in H^1(\Omega _{\boldsymbol{\alpha }})^2$ satisfies the adjoint system

3.6 $$\begin{aligned} \left\{ \begin{aligned}&\text {div}(\sigma (\boldsymbol{p}_{\boldsymbol{\alpha }})) = 2p\mu \text {div}\Big (\sigma _d({\boldsymbol{u}}_{\boldsymbol{\alpha }})(\sigma _d({\boldsymbol{u}}_{\boldsymbol{\alpha }}):\sigma _d({\boldsymbol{u}}_{\boldsymbol{\alpha }}))^{p/2-1}\Big ) \quad \text {in}\quad \Omega _{\boldsymbol{\alpha }}, \\&\sigma (\boldsymbol{p}_{\boldsymbol{\alpha }})\boldsymbol{n} = 2p\mu \Big (\sigma _d({\boldsymbol{u}}_{\boldsymbol{\alpha }})(\sigma _d({\boldsymbol{u}}_{\boldsymbol{\alpha }}):\sigma _d({\boldsymbol{u}}_{\boldsymbol{\alpha }}))^{p/2-1}\Big ) \boldsymbol{n} \quad \text {on}\quad \Gamma _{{\mathcal {N}}}\cup \partial \boldsymbol{\omega }_{\boldsymbol{\alpha }}, \\&\boldsymbol{p}_{\boldsymbol{\alpha }}= \boldsymbol{0}\quad \text {on}\quad \Gamma _{{\mathcal {D}}}. \end{aligned} \right. \end{aligned}$$

The respective variational formulation is given by

$$\begin{aligned} & \int _{\Omega _{\boldsymbol{\alpha }}}\sigma (\boldsymbol{p}_{\boldsymbol{\alpha }}):\varepsilon ({\boldsymbol{v}}) = \int _{\Omega _{\boldsymbol{\alpha }}}p(\sigma _d({\boldsymbol{u}}_{\boldsymbol{\alpha }}):\sigma _d({\boldsymbol{u}}_{\boldsymbol{\alpha }}))^{p/2-1}\sigma _d({\boldsymbol{u}}_{\boldsymbol{\alpha }}) \\ & : \sigma _d({\boldsymbol{v}}) \quad \forall \,{\boldsymbol{v}}\in {\boldsymbol{H}}_0(\Omega _{\boldsymbol{\alpha }}). \end{aligned}$$

Finally, the next proposition gives the shape derivative of the area of the deformed body ${{\,\mathrm{\mathcal {A}}\,}}(\Omega _{\boldsymbol{\alpha }})$. Since we have not seen an expression for this before, we shall provide the proof.

#### Proposition 3.7

The shape derivative of ${{\,\mathrm{\mathcal {A}}\,}}(\Omega _{\boldsymbol{\alpha }})$ is given by

3.7 $$\begin{aligned} \text {d}\,\,\!{{\,\mathrm{\mathcal {A}}\,}}(\Omega _{\boldsymbol{\alpha }})\langle \boldsymbol{\theta }_i\rangle= & \int _{\omega _i}\big (\det ({{\,\mathrm{{{\textbf {I}}}}\,}}+ \boldsymbol{\nabla }{\boldsymbol{u}}_{\boldsymbol{\alpha }}) - \sigma ({\boldsymbol{u}}_{\boldsymbol{\alpha }}): \varepsilon (\boldsymbol{q}_{\boldsymbol{\alpha }}) \nonumber \\ & \quad + \boldsymbol{f}\cdot \boldsymbol{q}_{\boldsymbol{\alpha }}\big )\big (\boldsymbol{\theta }\cdot \boldsymbol{n}\big ){{\,\mathrm{\text {d}\,\,\!{\boldsymbol{x}}}\,}}, \end{aligned}$$

where $i=1,\dots ,N$ and $\boldsymbol{q}_{\boldsymbol{\alpha }}\in H^1(\Omega _{\boldsymbol{\alpha }})^2$ satisfies the adjoint system

3.8 $$\begin{aligned} \left\{ \; \begin{aligned}&\text {div}(\sigma (\boldsymbol{q}_{\boldsymbol{\alpha }})) = \text {div}\big (\det ({{\,\mathrm{{{\textbf {I}}}}\,}}+\boldsymbol{\nabla }{\boldsymbol{u}}_{\boldsymbol{\alpha }})({{\,\mathrm{{{\textbf {I}}}}\,}}+\boldsymbol{\nabla }{\boldsymbol{u}}_{\boldsymbol{\alpha }})^{-\top }\big ) \quad \text {in}\quad \Omega _{\boldsymbol{\alpha }}, \\&\sigma (\boldsymbol{q}_{\boldsymbol{\alpha }})\boldsymbol{n} = \det ({{\,\mathrm{{{\textbf {I}}}}\,}}+\boldsymbol{\nabla }{\boldsymbol{u}}_{\boldsymbol{\alpha }})({{\,\mathrm{{{\textbf {I}}}}\,}}+\boldsymbol{\nabla }{\boldsymbol{u}}_{\boldsymbol{\alpha }})^{-\top }\boldsymbol{n} \quad \text {on}\quad \Gamma _{{\mathcal {N}}}\cup \partial \boldsymbol{\omega }_{\boldsymbol{\alpha }}, \\&\boldsymbol{q}_{\boldsymbol{\alpha }}= \boldsymbol{0}\quad \text {on}\quad \Gamma _{{\mathcal {D}}}. \end{aligned} \right. \end{aligned}$$

The respective variational formulation is given by

$$\begin{aligned} \int _{\Omega _{\boldsymbol{\alpha }}}\sigma (\boldsymbol{q}_{\boldsymbol{\alpha }}):\varepsilon ({\boldsymbol{v}})= & \int _{\Omega _{\boldsymbol{\alpha }}}\det ({{\,\mathrm{{{\textbf {I}}}}\,}}+\boldsymbol{\nabla }{\boldsymbol{u}}_{\boldsymbol{\alpha }})({{\,\mathrm{{{\textbf {I}}}}\,}}+\boldsymbol{\nabla }{\boldsymbol{u}}_{\boldsymbol{\alpha }})^{-\top } \\ & : \boldsymbol{\nabla }{\boldsymbol{v}}\quad \forall \,{\boldsymbol{v}}\in {\boldsymbol{H}}_0(\Omega _{\boldsymbol{\alpha }}). \end{aligned}$$

#### Proof

Since the proof does not depend on the particular choice of $1\le i \le N$, we will simplify our notation in accordance with $\boldsymbol{\theta }\equiv \boldsymbol{\theta }_i, \ \omega _i\equiv \omega ,\ \Omega _{\boldsymbol{\alpha }_\epsilon }\equiv \Omega _{\boldsymbol{\alpha }_{\epsilon _i}}$ and likewise $\boldsymbol{u}_{\boldsymbol{\alpha }_\epsilon }\equiv {\boldsymbol{u}}_{\boldsymbol{\alpha }_{\epsilon _i}}$.

For sufficiently small $\boldsymbol{\theta }\in W^{1,\infty }(\Omega _{\boldsymbol{\alpha }})^2$, a change of variables in the shape functional ${{\,\mathrm{\mathcal {A}}\,}}(\Omega _{\boldsymbol{\alpha }_\epsilon })$ yields

$$\begin{aligned} {{\,\mathrm{\mathcal {A}}\,}}(\Omega _{\boldsymbol{\alpha }_\epsilon })&= \int _{(\Omega _{\boldsymbol{\alpha }_\epsilon })_{\boldsymbol{u}_{\boldsymbol{\alpha }_\epsilon }}}{{\,\mathrm{\text {d}\,\,\!{\boldsymbol{x}}}\,}}\\&= \int _{\Omega _{\boldsymbol{\alpha }_\epsilon }}\det ({{\,\mathrm{{{\textbf {I}}}}\,}}+\boldsymbol{\nabla }\boldsymbol{u}_{\boldsymbol{\alpha }_\epsilon }){{\,\mathrm{\text {d}\,\,\!{\boldsymbol{x}}}\,}}\\&= \int _{\Omega _{\boldsymbol{\alpha }}}\det ({{\,\mathrm{{{\textbf {I}}}}\,}}+({{\,\mathrm{{{\textbf {I}}}}\,}}+\epsilon \boldsymbol{\nabla }\boldsymbol{\theta })^{-1}\boldsymbol{\nabla }{{\widehat{{\boldsymbol{u}}}}_{\boldsymbol{\alpha }_\epsilon }}) \det ({{\,\mathrm{{{\textbf {I}}}}\,}}+\epsilon \boldsymbol{\nabla }\boldsymbol{\theta }){{\,\mathrm{\text {d}\,\,\!{\boldsymbol{x}}}\,}}. \end{aligned}$$

By taking the derivative at $\epsilon =0$, we obtain

3.9 $$\begin{aligned} \begin{aligned} \text {d}\,\,\!{{\,\mathrm{\mathcal {A}}\,}}(\Omega _{\boldsymbol{\alpha }_\epsilon })\langle \boldsymbol{\theta }\rangle&= \int _{\Omega _{\boldsymbol{\alpha }}}\text {div}(\boldsymbol{\theta })\det ({{\,\mathrm{{{\textbf {I}}}}\,}}+ \boldsymbol{\nabla }{\boldsymbol{u}}_{\boldsymbol{\alpha }}){{\,\mathrm{\text {d}\,\,\!{\boldsymbol{x}}}\,}}\\&+ \int _{\Omega _{\boldsymbol{\alpha }}}\det ({{\,\mathrm{{{\textbf {I}}}}\,}}+\boldsymbol{\nabla }{\boldsymbol{u}}_{\boldsymbol{\alpha }}){{\,\mathrm{\text {tr}}\,}}({{\,\mathrm{{{\textbf {I}}}}\,}}+\boldsymbol{\nabla }{\boldsymbol{u}}_{\boldsymbol{\alpha }})^{-1}(\boldsymbol{\nabla }\dot{{\boldsymbol{u}}}_{\boldsymbol{\alpha }}-\boldsymbol{\nabla }\boldsymbol{\theta }\boldsymbol{\nabla }{\boldsymbol{u}}_{\boldsymbol{\alpha }})){{\,\mathrm{\text {d}\,\,\!{\boldsymbol{x}}}\,}}\\&=\int _{\Omega _{\boldsymbol{\alpha }}}\text {div}(\boldsymbol{\theta })\det ({{\,\mathrm{{{\textbf {I}}}}\,}}+ \boldsymbol{\nabla }{\boldsymbol{u}}_{\boldsymbol{\alpha }}){{\,\mathrm{\text {d}\,\,\!{\boldsymbol{x}}}\,}}\\&+ \int _{\Omega _{\boldsymbol{\alpha }}}\det ({{\,\mathrm{{{\textbf {I}}}}\,}}+\boldsymbol{\nabla }{\boldsymbol{u}}_{\boldsymbol{\alpha }})({{\,\mathrm{{{\textbf {I}}}}\,}}+\boldsymbol{\nabla }{\boldsymbol{u}}_{\boldsymbol{\alpha }})^{-\top }:\boldsymbol{\nabla }\dot{{\boldsymbol{u}}}_{\boldsymbol{\alpha }}{{\,\mathrm{\text {d}\,\,\!{\boldsymbol{x}}}\,}}\\&-\int _{\Omega _{\boldsymbol{\alpha }}}\det ({{\,\mathrm{{{\textbf {I}}}}\,}}+\boldsymbol{\nabla }{\boldsymbol{u}}_{\boldsymbol{\alpha }})({{\,\mathrm{{{\textbf {I}}}}\,}}+\boldsymbol{\nabla }{\boldsymbol{u}}_{\boldsymbol{\alpha }})^{-\top }:(\boldsymbol{\nabla }\boldsymbol{\theta }\boldsymbol{\nabla }{\boldsymbol{u}}_{\boldsymbol{\alpha }}){{\,\mathrm{\text {d}\,\,\!{\boldsymbol{x}}}\,}}. \end{aligned} \end{aligned}$$

Next, we formulate the variational identity for the adjoint state $\boldsymbol{q}_{\boldsymbol{\alpha }}\in {\boldsymbol{H}}_0(\Omega _{\boldsymbol{\alpha }})$ as follows:

3.10 $$\begin{aligned} \int _{\Omega _{\boldsymbol{\alpha }}} \sigma (\boldsymbol{q}_{\boldsymbol{\alpha }}):\varepsilon ({\boldsymbol{v}}){{\,\mathrm{\text {d}\,\,\!{\boldsymbol{x}}}\,}} & = \int _{\Omega _{\boldsymbol{\alpha }}}\det ({{\,\mathrm{{{\textbf {I}}}}\,}}+\boldsymbol{\nabla }{\boldsymbol{u}}_{\boldsymbol{\alpha }})({{\,\mathrm{{{\textbf {I}}}}\,}}+\boldsymbol{\nabla }{\boldsymbol{u}}_{\boldsymbol{\alpha }})^{-\top } \nonumber \\ & :\boldsymbol{\nabla }{\boldsymbol{v}}{{\,\mathrm{\text {d}\,\,\!{\boldsymbol{x}}}\,}}\quad \forall \,{\boldsymbol{v}}\in {\boldsymbol{H}}_0(\Omega _{\boldsymbol{\alpha }}). \end{aligned}$$

One readily checks that the variational formulation (3.10) corresponds to the boundary value problem (3.8). By taking $\dot{{\boldsymbol{u}}}_{\boldsymbol{\alpha }}$ as test function in (3.10) and $\boldsymbol{q}_{\boldsymbol{\alpha }}$ as test function in (3.3), we conclude

$$\begin{aligned} \int _{\Omega _{\boldsymbol{\alpha }}}\det ({{\,\mathrm{{{\textbf {I}}}}\,}}+\boldsymbol{\nabla }{\boldsymbol{u}}_{\boldsymbol{\alpha }})({{\,\mathrm{{{\textbf {I}}}}\,}}+&\boldsymbol{\nabla }{\boldsymbol{u}}_{\boldsymbol{\alpha }})^{-\top }:\boldsymbol{\nabla }\dot{{\boldsymbol{u}}}_{\boldsymbol{\alpha }}{{\,\mathrm{\text {d}\,\,\!{\boldsymbol{x}}}\,}}\\&= \int _{\Omega _{\boldsymbol{\alpha }}}\big (\mathbb {C}:(\boldsymbol{\nabla }\boldsymbol{\theta }\boldsymbol{\nabla }{\boldsymbol{u}}_{\boldsymbol{\alpha }}) : \boldsymbol{\nabla }\boldsymbol{q}_{\boldsymbol{\alpha }}\\&\quad + \mathbb {C}:\boldsymbol{\nabla }{\boldsymbol{u}}_{\boldsymbol{\alpha }}:(\boldsymbol{\nabla }\boldsymbol{\theta }\boldsymbol{\nabla }\boldsymbol{q}_{\boldsymbol{\alpha }})\big ){{\,\mathrm{\text {d}\,\,\!{\boldsymbol{x}}}\,}}\\&-\int _{\Omega _{\boldsymbol{\alpha }}}\big (\text {div}(\boldsymbol{\theta })\sigma ({\boldsymbol{u}}_{\boldsymbol{\alpha }}) : \varepsilon (\boldsymbol{q}_{\boldsymbol{\alpha }}) \\&\quad - \text {div}(\boldsymbol{f}\otimes \boldsymbol{\theta })\cdot \boldsymbol{q}_{\boldsymbol{\alpha }}\big ){{\,\mathrm{\text {d}\,\,\!{\boldsymbol{x}}}\,}}. \end{aligned}$$

Thus, we can rewrite (3.9) as

3.11 $$\begin{aligned} \begin{aligned} \text {d}\,\,\!{{\,\mathrm{\mathcal {A}}\,}}(\Omega _{\boldsymbol{\alpha }_\epsilon })\langle \boldsymbol{\theta }\rangle&=\int _{\Omega _{\boldsymbol{\alpha }}}\text {div}(\boldsymbol{\theta })\det ({{\,\mathrm{{{\textbf {I}}}}\,}}+ \boldsymbol{\nabla }{\boldsymbol{u}}_{\boldsymbol{\alpha }}){{\,\mathrm{\text {d}\,\,\!{\boldsymbol{x}}}\,}}\\&+ \int _{\Omega _{\boldsymbol{\alpha }}}\big (\mathbb {C}:(\boldsymbol{\nabla }\boldsymbol{\theta }\boldsymbol{\nabla }{\boldsymbol{u}}_{\boldsymbol{\alpha }}): \boldsymbol{\nabla }\boldsymbol{q}_{\boldsymbol{\alpha }}+ \mathbb {C}:\boldsymbol{\nabla }{\boldsymbol{u}}_{\boldsymbol{\alpha }}:(\boldsymbol{\nabla }\boldsymbol{\theta }\boldsymbol{\nabla }\boldsymbol{q}_{\boldsymbol{\alpha }})\big ){{\,\mathrm{\text {d}\,\,\!{\boldsymbol{x}}}\,}}\\&-\int _{\Omega _{\boldsymbol{\alpha }}}\big (\text {div}(\boldsymbol{\theta })\sigma ({\boldsymbol{u}}_{\boldsymbol{\alpha }}): \varepsilon (\boldsymbol{q}_{\boldsymbol{\alpha }}) - \text {div}(\boldsymbol{f}\otimes \boldsymbol{\theta })\cdot \boldsymbol{q}_{\boldsymbol{\alpha }}\big ){{\,\mathrm{\text {d}\,\,\!{\boldsymbol{x}}}\,}}\\&-\int _{\Omega _{\boldsymbol{\alpha }}}\det ({{\,\mathrm{{{\textbf {I}}}}\,}}+\boldsymbol{\nabla }{\boldsymbol{u}}_{\boldsymbol{\alpha }})({{\,\mathrm{{{\textbf {I}}}}\,}}+\boldsymbol{\nabla }{\boldsymbol{u}}_{\boldsymbol{\alpha }})^{-\top }:(\boldsymbol{\nabla }\boldsymbol{\theta }\boldsymbol{\nabla }{\boldsymbol{u}}_{\boldsymbol{\alpha }}){{\,\mathrm{\text {d}\,\,\!{\boldsymbol{x}}}\,}}. \end{aligned} \end{aligned}$$

In tensor notation, (3.11) can be rewritten as

$$\begin{aligned} \text {d}\,\,\!{{\,\mathrm{\mathcal {A}}\,}}(\Omega _{\boldsymbol{\alpha }_\epsilon })\langle \boldsymbol{\theta }\rangle = \int _{\Omega _{\boldsymbol{\alpha }}} {{\,\mathrm{{{\textbf {T}}}}\,}}:\boldsymbol{\nabla }\boldsymbol{\theta }{{\,\mathrm{\text {d}\,\,\!{\boldsymbol{x}}}\,}}, \end{aligned}$$

where

$$\begin{aligned} {{\,\mathrm{{{\textbf {T}}}}\,}}&= \big (\det ({{\,\mathrm{{{\textbf {I}}}}\,}}+ \boldsymbol{\nabla }{\boldsymbol{u}}_{\boldsymbol{\alpha }})- \sigma ({\boldsymbol{u}}_{\boldsymbol{\alpha }}) : \varepsilon (\boldsymbol{q}_{\boldsymbol{\alpha }}) + \boldsymbol{f}\cdot \boldsymbol{q}_{\boldsymbol{\alpha }}\big ){{\,\mathrm{{{\textbf {I}}}}\,}}\\&\quad + \mathbb {C}:(\boldsymbol{\nabla }{\boldsymbol{u}}_{\boldsymbol{\alpha }}\boldsymbol{q}_{\boldsymbol{\alpha }}^\top ) + \mathbb {C}:(\boldsymbol{\nabla }\boldsymbol{q}_{\boldsymbol{\alpha }}{\boldsymbol{u}}_{\boldsymbol{\alpha }}^\top ) \\&\quad - \det ({{\,\mathrm{{{\textbf {I}}}}\,}}+\boldsymbol{\nabla }{\boldsymbol{u}}_{\boldsymbol{\alpha }})({{\,\mathrm{{{\textbf {I}}}}\,}}+\boldsymbol{\nabla }{\boldsymbol{u}}_{\boldsymbol{\alpha }})^{-\top }\boldsymbol{\nabla }{\boldsymbol{u}}^\top . \end{aligned}$$

Thus, the application of [Laurain and Sturm (2016), Theorem 3.1] under the assumption that $\boldsymbol{\theta }= (\boldsymbol{\theta }\cdot \boldsymbol{n})\boldsymbol{n}$ on $\partial \boldsymbol{\omega }_{\boldsymbol{\alpha }}$ yields

3.12 $$\begin{aligned} \text {d}\,\,\!{{\,\mathrm{\mathcal {A}}\,}}(\Omega _{\boldsymbol{\alpha }_\epsilon })\langle \boldsymbol{\theta }\rangle = \int _{\partial \boldsymbol{\omega }_{\boldsymbol{\alpha }}} {{\,\mathrm{{{\textbf {T}}}}\,}}\boldsymbol{n}\cdot \boldsymbol{n}(\boldsymbol{\theta }\cdot \boldsymbol{n}){{\,\mathrm{\text {d}\,\,\!\boldsymbol{s}}\,}}. \end{aligned}$$

By means of Green’s formula to (3.10), we conclude in view of the boundary conditions in (2.1) that

3.13 $$\begin{aligned} \sigma (\boldsymbol{q}_{\boldsymbol{\alpha }})\boldsymbol{n} = \det ({{\,\mathrm{{{\textbf {I}}}}\,}}+\boldsymbol{\nabla }{\boldsymbol{u}}_{\boldsymbol{\alpha 
}})({{\,\mathrm{{{\textbf {I}}}}\,}}+\boldsymbol{\nabla }{\boldsymbol{u}}_{\boldsymbol{\alpha }})^{-\top 
}\boldsymbol{n} \quad \text {and}\quad \sigma ({\boldsymbol{u}}) \boldsymbol{n} = \boldsymbol{0}\quad \text {on}\quad \partial \boldsymbol{\omega }_{\boldsymbol{\alpha }}. \end{aligned}$$

The application of (3.13) to (3.12) yields the desired expression, thus completing the proof. $\square$

## Numerical realization

In this part of the article, we develop the numerical method for solving the shape optimization problems under consideration. We employ both, the gradient descent method as such and its combination with a genetic algorithm. We aim at optimizing a quadratic piece of skin, in which the cuts are modelled as slits with locations in periodic cells within the domain. We present the outcomes of the gradient descent method in the case of stretching the skin in one axis direction and the outcomes of the combination of a genetic algorithm and the gradient descent method in the case of stretching in two axes directions.

### Optimization algorithm

To solve the optimal cut layout problems (2.4)–(2.6), we employ the classical gradient descent method

4.1 $$\begin{aligned} \boldsymbol{\alpha }_{n+1} = \boldsymbol{\alpha }_{n} - \gamma _n{{\,\mathrm{{{\textbf {D}}}\!}\,}}{{\,\mathrm{\mathcal {F}}\,}}(\Omega _{\boldsymbol{\alpha }_n})\langle \boldsymbol{\Theta }\rangle , \end{aligned}$$

where $\mathcal {F}(\Omega _{\boldsymbol{\alpha }_n})$ is one of the considered shape functionals, ${{\,\mathrm{{{\textbf {D}}}\!}\,}}{{\,\mathrm{\mathcal {F}}\,}}(\Omega _{\boldsymbol{\alpha }_n})\langle \boldsymbol{\Theta }\rangle$ is the shape gradient calculated in accordance with Proposition 3.5 for the perturbation field $\boldsymbol{\Theta }= [\boldsymbol{\theta }_1, \dots , \boldsymbol{\theta }_N]$ defined by (3.1). The step size $\gamma _n$ is found by a quadratic line search with at most five iterations. Note that in the case of maximization of the area of the deformed body, we just switch the sign in front of the shape functional to cast it into a minimization problem.

Numerical experiments have shown that the shape optimization problem under consideration has a low sensitivity and many local minima, which in some configurations makes the solution of the problem by the gradient descent method significantly more difficult, even when using accelerated variants such as the quasi-Newton or the Nesterov scheme, see Fletcher (1980); Nesterov (2018) for example. Therefore, inspired by the successful results of combining global optimization methods with continuous optimization in Harbrecht and Loos (2016) for a similar shape optimization problem, we shall address the application of a genetic algorithm, cf. Haupt and Haupt (2004).

A genetic algorithm is a stochastic method used to solve optimization problems. It lacks the precision of gradient-based methods because it does not study the function to be minimized. It evaluates the objective functional (*fitness*) for the optimization variables (*individuals*). The algorithm runs as follows: an initial population of individuals evolves over multiple generations through the simulated genetic operations of *mutation* and *crossover*. The fittest individuals survive and reproduce, advancing the population. The initial population consists of $M$ individuals, obtained by a specified heuristics or generated randomly. To transition from one generation to the next, we follow the steps:

1. The current population (*parents*) mutates and produces a generation of *children*.
2. The *elite* is selected from the generations of parents and children based on the fitness.
3. The elite is crossed to produce the new children.
4. The new elite replaces the old population.

As a mutation, we use a gradient descent step (4.1) with step size $\gamma _n=\Vert {{\,\mathrm{{{\textbf {D}}}\!}\,}}{{\,\mathrm{\mathcal {F}}\,}}(\Omega _{\boldsymbol{\alpha }_n})\langle \boldsymbol{\Theta }\rangle \Vert _2^{-1}$. Crossover children are created by intersecting the vectors of a pair of parents: we randomly choose two parents (here: $p_1$ and $p_2$) and a cut position, then we exchange the components of both parents and create two children (here: $c_1$ and $c_2$). For example:

$$\begin{aligned} \begin{array}{l c l} \begin{array}{c} p_1 = [1,2,3,4,5,6,7] \\ p_2 = [a,b,c,d,e,f,g] \end{array} \begin{array}{c} {\longrightarrow } \end{array} \begin{array}{c} c_1 = [1,2,3,4,e,f,g] \\ c_2 = [a,b,c,d,5,6,7] \end{array} \end{array} \end{aligned}$$

Identical individuals are not allowed in the population. Then, at some stage, the fittest individual no longer changes and displaces all others in the population by the crossover and elite choice. Once it has displaced all the rest, the algorithm naturally stops. Note that the most computationally expensive part of the above algorithm is the computation of the solution of the direct problem and the calculation of the shape gradient. This, however, can be easily parallelized.

### Computational setup

The domain $\Omega = (0,1)^2$, which represents the piece of skin, is chosen as the unite square. We divide the domain into 3$\times$3 equal blocks, each of which being a copy of the others. Inside these blocks, we introduce a grid of 4$\times$4 quadratic cells of edge size $1/12$. In the centre of each cell, we put an ellipse-shaped cut with semi-axes $0.75/12$ and $0.05/12$. Thus, we get ${\boldsymbol{\omega }}_{\boldsymbol{\alpha }}$ with 144 cuts in all, but only $N=16$ design variables (the rotation angles of the cuts), compare Fig. 1. We do so in order to improve the optimization results, since each of the components $\boldsymbol{\alpha }$ defines nine cuts at once which increases the sensitivity of the functional with respect to a change in the design variables.

**Fig. 3:**
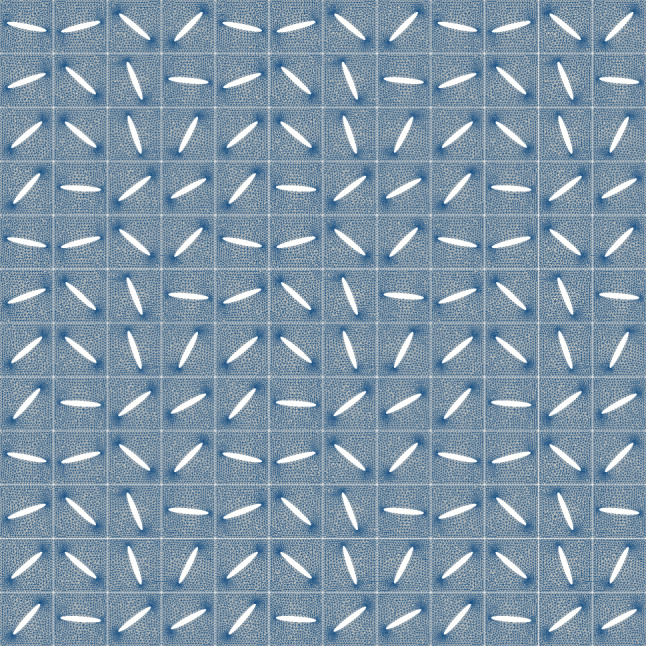
Skin grafting model with a random configuration of cuts. The finite element mesh consists of roughly 165,000 finite elements

**Fig. 4:**
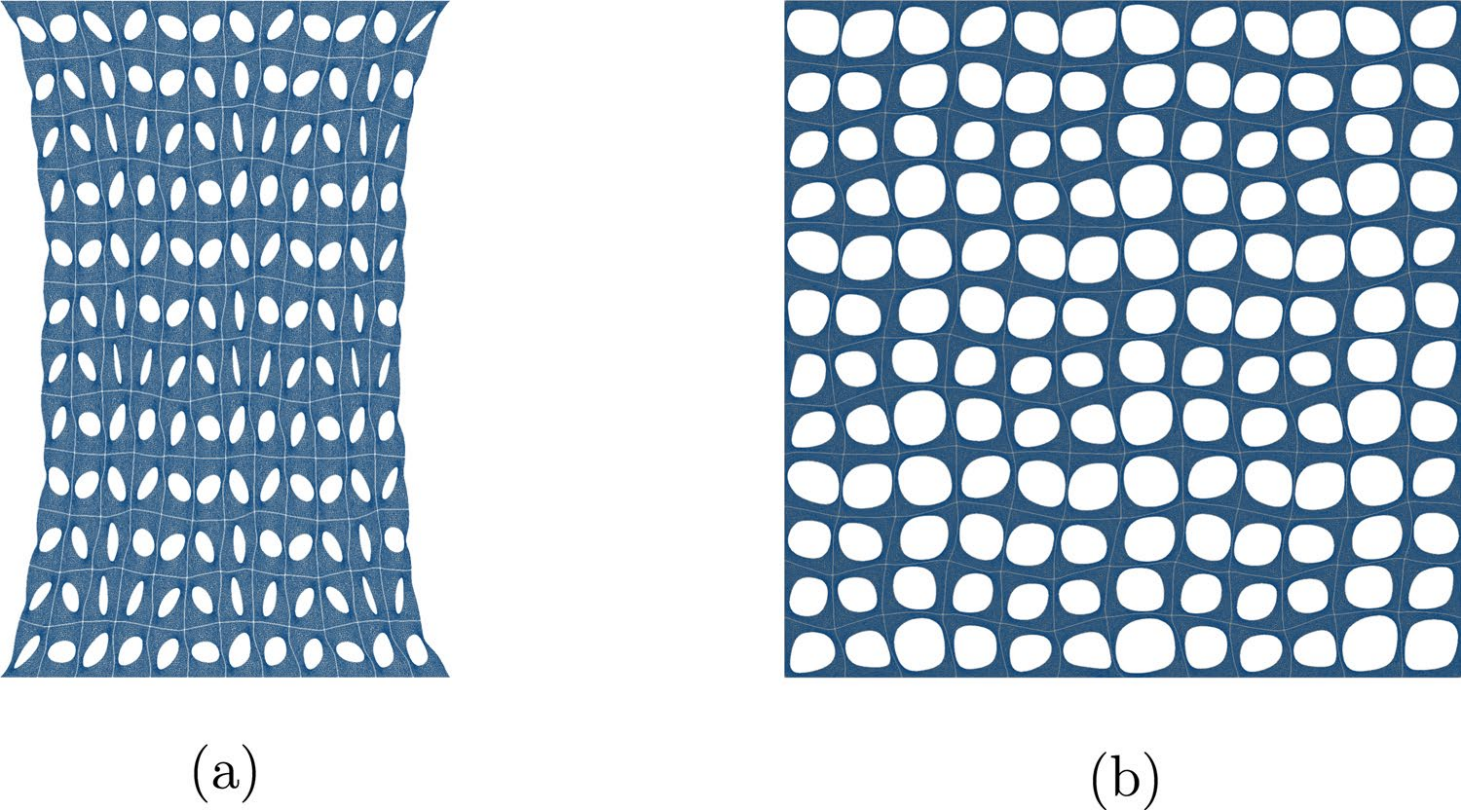
Stretched skin for the random configuration of cuts: (a) single axis stretching, (b) bi-axial stretching

For solving the equations of linear elasticity on the respective layout, we apply the finite element method as provided by the finite element solver FreeFem++, see Hecht (2012). The material parameters are set as proposed in the review article (Kalra et al. 2016): the Young modulus is $E=50$ MPa and the Poisson is ratio $\nu =0.48$. Since the finite element mesh is automatically adapted towards the current geometry during the optimization process, it is sufficient to say that the finite element mesh size varies from $h_\text {min} = 6.5\cdot 10^{-4}$ to $h_\text {min} = 7.5\cdot 10^{-3}$, which leads to a number of about 165,000 finite elements. An illustration of the finite element mesh in case of a random configuration can be found in Fig. 3.

We consider two stress models. The first one is stretching in one axial direction, i.e. $\Gamma _{{\mathcal {D}}}$ consists of two opposite boundaries of the square $\Omega$, on which there is a Dirichlet condition, and the remaining boundaries $\Gamma _{{\mathcal {N}}}$ are free. The second one is the stretching in both axial directions, i.e. a Dirichlet boundary condition is prescribed at the whole boundary $\partial \Omega = \Gamma _{{\mathcal {D}}}$. In both cases, we set $\boldsymbol{g}=0.25\boldsymbol{n}$ and $\boldsymbol{f}=\boldsymbol{0}$. Note that, with this setup, the solution of the equations of linear elasticity will admit singularities in the vertices of the square. A visualization of the stretched skin in case of the random configuration from Fig. 3 can be found in Fig. 4.

### Numerical results: stretching in one axial direction

**Fig. 5:**
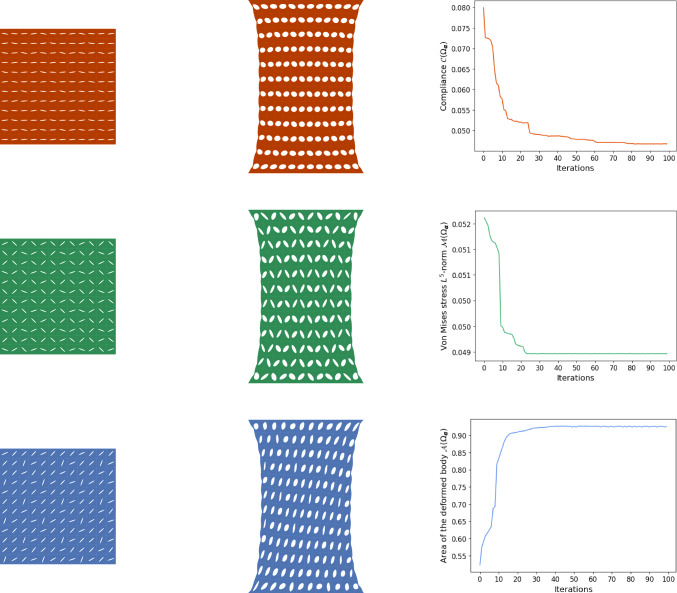
Stretching in one axial direction. Final cut configuration (left), deformed skin (middle), and convergence history (right): first row—compliance $\mathcal {C}(\Omega _{\boldsymbol{\alpha }})$, second row—$L^5$-norm of the von Mises stress ${{\,\mathrm{\mathcal {M}}\,}}(\Omega _{\boldsymbol{\alpha }})$, third row—area of the deformed body ${{\,\mathrm{\mathcal {A}}\,}}(\Omega _{\boldsymbol{\alpha }})$

In the case of stretching in one axial direction, the gradient descent method provides satisfactory results. The initial configuration is the same for all experiments and the one presented in Fig. 3. A total of 100 iterations is performed, and the outcomes obtained are presented in Fig. 5. Note that, in all our subsequent experiments, we have chosen $p=5$ when minimizing the $L^p$-norm of the von Mises stress.

When minimizing the compliance $\mathcal {C}(\Omega _{\boldsymbol{\alpha }})$, the optimal design is a horizontal arrangement of the cuts. More precisely, all cuts are perpendicular to the stretch direction, which is a reasonable configuration, see the first row in the Fig. 5. It allows for the largest possible opening of the cuts, which makes the stretching process easier. The deformation in this situation is characterized by a minimum amount of work. However, the skin in the area between the cuts is quite thin, which increases its stress. Therefore, it is not surprising that, in order to decrease the stress intensity, it is necessary to find a better balance between stretching easiness and maintaining sufficient thickness in the areas between the cuts. Thus, when minimizating the $L^5$-norm of the von Mises stress ${{\,\mathrm{\mathcal {M}}\,}}(\Omega _{\boldsymbol{\alpha }})$, the optimal design is a zigzag configuration, compare the second row in Fig. 5.

**Fig. 6:**
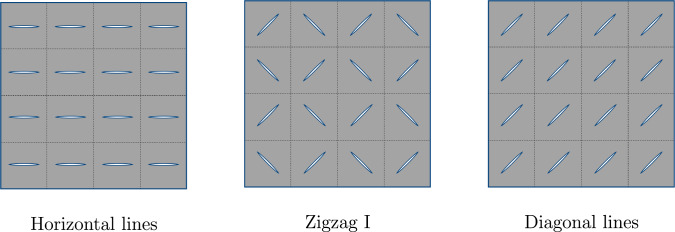
Illustration of the optimal designs in case of stretching the skin in one axial direction

Finally, in the case of the maximization of the area of the stretched skin, i.e. when considering the shape functional ${{\,\mathrm{\mathcal {A}}\,}}(\Omega _{\boldsymbol{\alpha }})$, we on the one hand maximize the area of the entire deformed body ${{\,\mathrm{\mathcal {A}}\,}}(\Omega _{{\boldsymbol{u}}_{\boldsymbol{\alpha }}})$ and on the other hand minimize the size of the deformed cuts ${{\,\mathrm{\mathcal {A}}\,}}(({\boldsymbol{\omega }}_{\boldsymbol{\alpha }})_{{\boldsymbol{u}}_{\boldsymbol{\alpha }}})$. Therefore, the logical result is a diagonal configuration of the cuts, see the third row in Fig. 5. Then, the contraction of the body perpendicular to stretching and the deformation of the cuts are in balance.

The optimal configurations found by the optimization process are summarized in Fig. 6 without computational noise in the outcomes caused by the simulation process.

### Numerical results: stretching in two axial directions

**Fig. 7:**
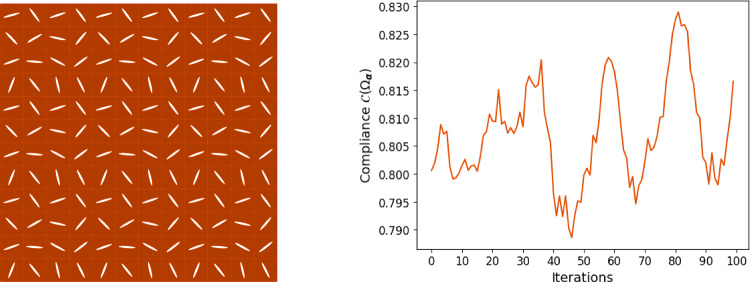
Result of the gradient descent method for the compliance $\mathcal {C}(\Omega _{\boldsymbol{\alpha }})$ when we stretch the skin in two axial directions: Final cut configuration (left) and convergence history (right)

In the case of stretching the skin in both axial directions, solely using the gradient descent method is not successful since we get stuck in one of the many local minima. We refer the reader to Fig. 7 where we present the respective result in case of the minimization of the compliance functional.

**Fig. 8:**
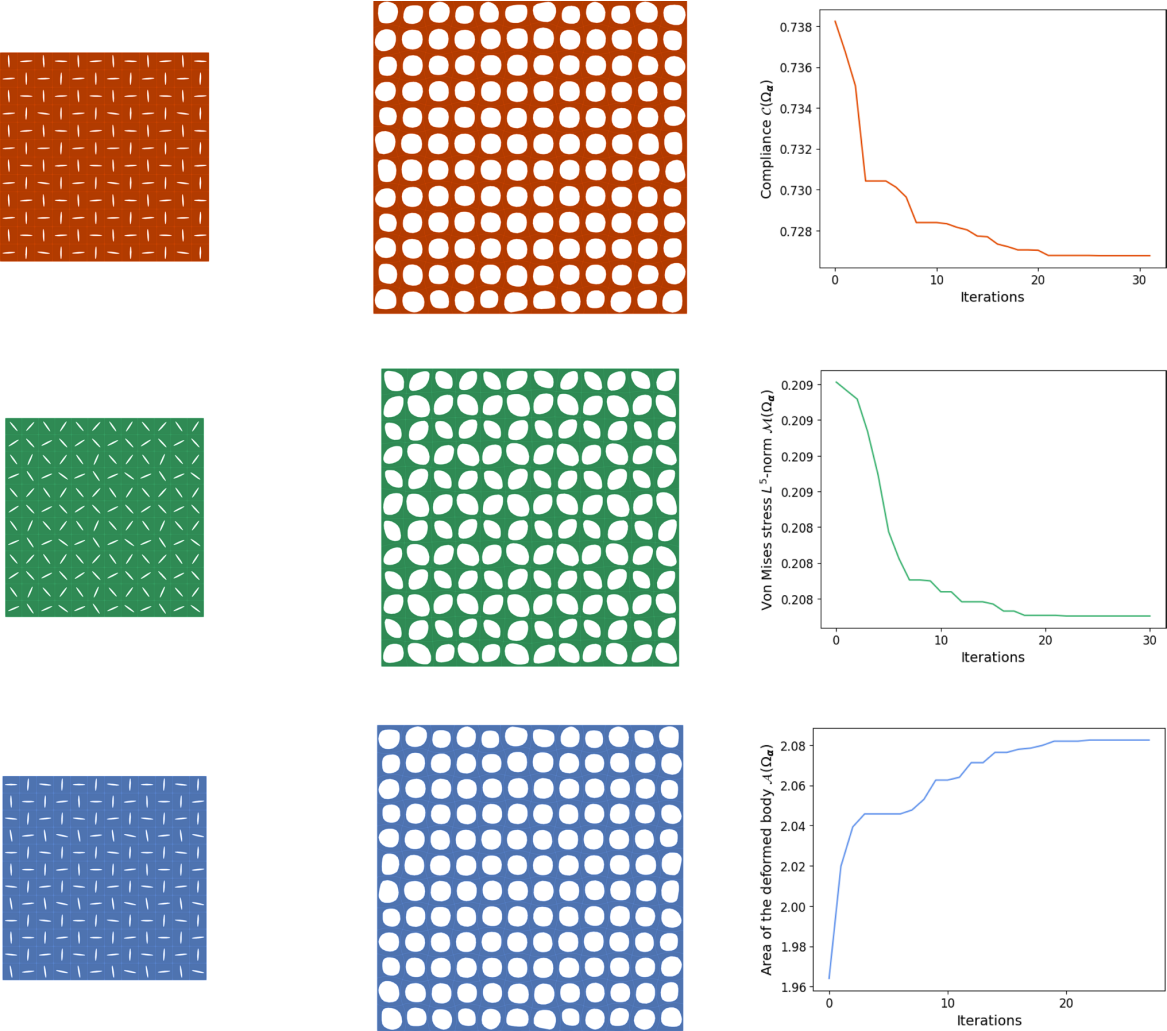
Stretching in two axial directions. Final cut configuration (left), deformed skin (middle) and convergence history(right): first row—compliance $\mathcal {C}(\Omega _{\boldsymbol{\alpha }})$, second row—$L^5$-norm of the von Mises stress ${{\,\mathrm{\mathcal {M}}\,}}(\Omega _{\boldsymbol{\alpha }})$, third row—area of the deformed body ${{\,\mathrm{\mathcal {A}}\,}}(\Omega _{\boldsymbol{\alpha }})$

However, the combination of the gradient descent method with the genetic algorithm gives reasonable results. The initial population consisted of 150 random individuals as specified in Fig. 5. It took about 30 iterations for each functional to obtain a single elite individual. The results can be found in Fig. 8. The convergence histories represent the values of the objective functional for the fittest individual in the population during the algorithm’s execution.

In the case of compliance minimization $\mathcal {C}(\Omega _{\boldsymbol{\alpha }})$, we obtained a configuration that switches between horizontal and vertical cuts, see the first row in Fig. 8. This configuration is remarkable because it gives the skin auxetic properties. Unlike conventional structures, auxetic ones expand perpendicular to the stretching axes rather than shrinking. This results in a negative Poisson ratio. With this configuration, the work required to deform the body is minimized. We get also the same configuration when maximizing the area of the deformed body ${{\,\mathrm{\mathcal {A}}\,}}(\Omega _{\boldsymbol{\alpha }})$.^1^

**Fig. 9:**
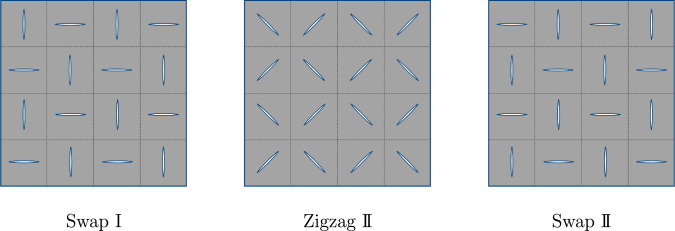
Illustration of the optimal designs in case of stretching the skin in two axial directions

In the case of stretching in two axial directions, the area of the body without taking into account the cuts ${{\,\mathrm{\mathcal {A}}\,}}(\Omega _{{\boldsymbol{u}}_{\boldsymbol{\alpha }}})$ is always the same, since we specify the deformation of the entire boundary, but the area of the cuts themselves changes. Therefore, this problem of maximization of the area of the skin amounts to minimizing the area ${{\,\mathrm{\mathcal {A}}\,}}\big (({\boldsymbol{\omega }}_{\boldsymbol{\alpha }})_{{\boldsymbol{u}}_{\boldsymbol{\alpha }}}\big )$ of the cuts after deformation. Therefore, it is optimal to deform the elliptical cuts into circles, which indeed can be achieved with the swapping pattern.

Finally, when minimizing the $L^5$-norm of the von Mises stress ${{\,\mathrm{\mathcal {M}}\,}}(\Omega _{\boldsymbol{\alpha }})$, we obtained the same zigzag pattern as when we stretch in one axial direction. This is reasonable since in the present situation it is important to avoid thinning of the skin between the cuts where the stress is highest.

The optimal configurations found by the optimization process are again summarized in Fig. 9 without computational noise in the outcomes caused by the simulation process.

## Conclusion

In this article, we considered the problem of optimizing the layout of the cut configuration for skin grafting within the framework of linear elasticity. The cuts are located in periodic cells and are specified by the angle of rotation. We formulated the optimization problem with three objective functionals: the compliance, the $L^p$-norm of the von Mises stress, and the area of the deformed body. For the solution of each of these optimization problems, we established an existence result. By using shape calculus, we derived the corresponding shape gradients with respect to the cut configuration and implemented a numerical approach based on gradient descent. The combination of this approach with a genetic algorithm avoids getting stuck in one of the numerous local minima. We obtained reasonable numerical results in the case of stretching the skin in one and two axial directions.

The presented approach offers a mathematically solid and computationally feasible tool for the optimization in the context of skin grafting. It, however, also opens up several directions for future research. Extending the mathematical model to include hyperelastic behaviour would better capture the non-linear mechanical properties of the skin. Further, allowing more general cut geometries could enhance the practical relevance of the simulations. Finally, the formulation of optimization strategies that explicitly promote auxetic behaviour could offer promising avenues for improving the outcomes of skin grafts.

## Data Availability

The results presented in this article can be replicated by implementing the data structures and algorithms presented in this article. The objective functionals and their shape gradients can be used as described and only require the inputs specified in the problem’s description.

## Footnotes

1 By taking a closer look to Fig. 8, one readily infers that the swap pattern is shifted, compare also Fig. 9. But for symmetry reasons, both patterns represent minimizers with identical values in the respective objective functional.
